# Post-CDK4/6 inhibitor therapy in HR+/HER2– advanced breast cancer: expanding options and persisting uncertainties

**DOI:** 10.37349/etat.2026.1002400

**Published:** 2026-09-11

**Authors:** Anne-Murielle Hollinger, Ruben Bill

**Affiliations:** Fondazione Policlinico Universitario Agostino Gemelli IRCCS, Italy; Department of Medical Oncology, Inselspital, Bern University Hospital, University of Bern, 3010 Bern, Switzerland

**Keywords:** HR-positive breast cancer, HER2-negative breast cancer, CDK4/6 inhibitors, endocrine resistance, endocrine therapy, PI3K/AKT/mTOR pathway, *ESR1* mutation, *PIK3CA* mutation

## Abstract

Endocrine treatment in combination with CDK4/6 inhibitors represents the mainstay of first-line palliative treatment in HR+/HER2– breast cancer. The treatment landscape after progression on CDK4/6 inhibitors has evolved into a complex and rapidly evolving field, driven by novel endocrine and targeted agents, new combination therapies, and increasing implementation of biomarker-directed approaches. Recent randomized trials have shown that new therapeutic strategies significantly prolong progression-free survival in this setting, with some treatments reaching notable numerical improvements. Translating these options into routine clinical practice remains challenging due to heterogeneous trial designs, lack of head-to-head comparisons, suboptimal comparator treatments, and limited overall survival data. While the increasing implementation of biomarkers holds the potential for more personalized treatments, their clinical utility depends on their mechanistic and contextual biological relevance, methodological reliability of detection, and proven predictive value in clinical trials. Based on the current evidence, optimal treatment selection and sequencing in the post-CDK4/6 inhibitor setting remain insufficiently defined, and optimization of biomarker-guided treatment requires further mechanistic understanding and methodological refinement. This narrative review provides an overview of the most recent randomized clinical trial evidence shaping current clinical practice and discusses persistent uncertainties regarding its implementation in routine care.

## Introduction

Breast cancer is the most frequently diagnosed cancer and remains the leading cause of cancer mortality in females globally [1]. Prognosis and treatment of advanced breast cancer (ABC) depend on the respective subtype, with hormone-receptor positive (HR+), HER2-negative (HER2–) disease representing the most prevalent one [2]. The introduction of CDK4/6 inhibitors (CDK4/6i) in combination with endocrine therapy (ET) has led to substantial therapeutic advances in HR+/HER2– locally advanced inoperable and metastatic breast cancer (here collectively referred to as ABC). As a result, these agents have become a mainstay of first-line palliative therapy and have more recently been incorporated into the adjuvant setting [3–11]. Despite these advances, the vast majority of patients receiving CDK4/6i therapy in the advanced disease setting inevitably experience disease progression, necessitating subsequent lines of therapy. Therapeutic decision-making after progression on CDK4/6i-based first-line therapy, however, remains highly complex. An expanding variety of therapeutic options, driven by positive results from recent clinical trials, challenges treating physicians to select the optimal, personalized treatment strategy.

**Figure 1:**
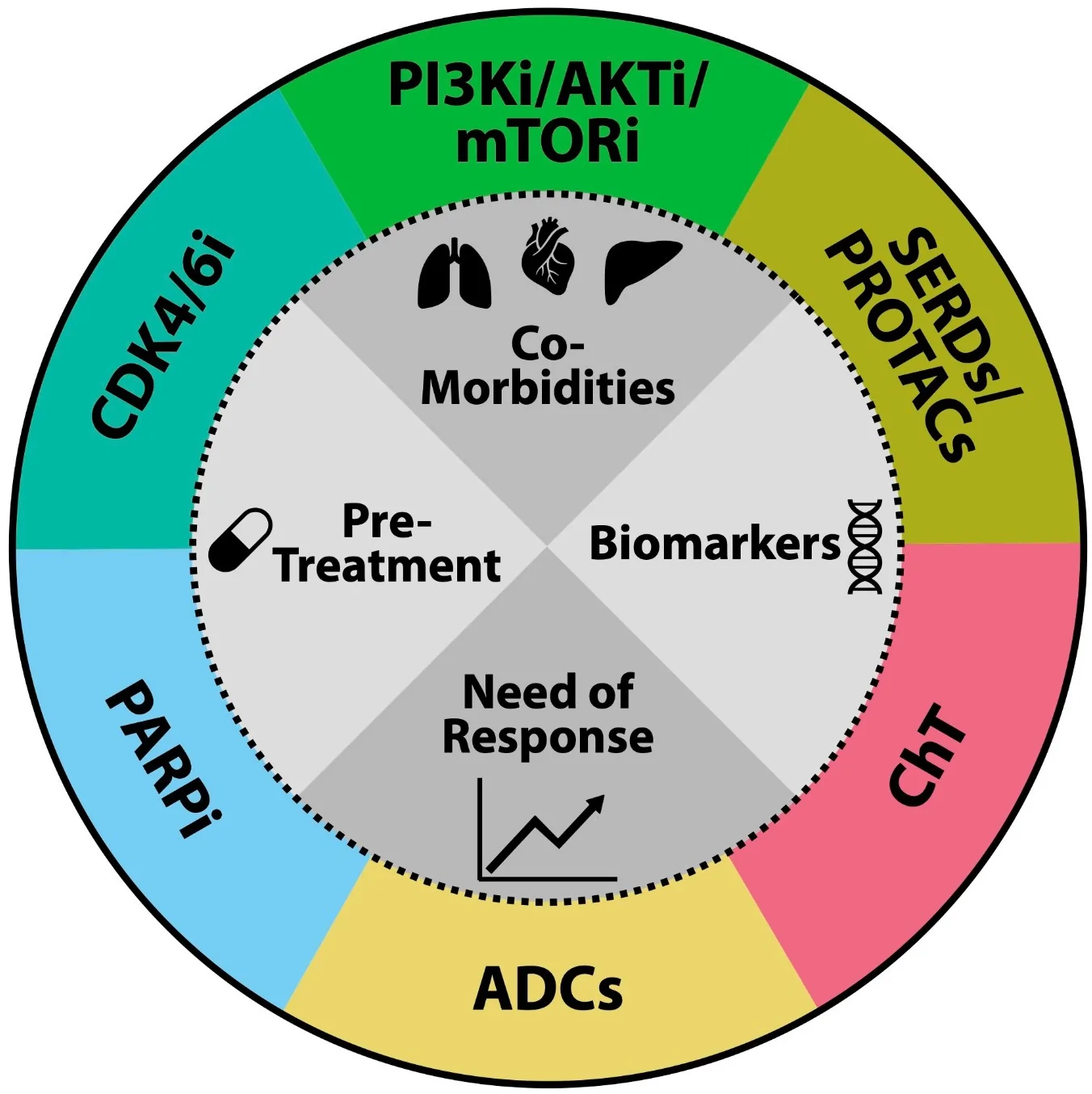
**Determinants influencing treatment selection after progression on CDK4/6i and approved drug classes available for HR+/HER2– advanced breast cancer.** ADCs: antibody-drug conjugates; AKTi: AKT inhibitor; ChT: chemotherapy; PI3Ki: phosphoinositide 3-kinase inhibitor; mTORi: mechanistic target of rapamycin inhibitor; PARPi: Poly(ADP-ribose)-polymerase inhibitor; PROTACs: proteolysis-targeting chimeras. Icons provided by Apple Keynote.

According to current guidelines, treatment after progression on ET in combination with a CDK4/6i should be individualized according to a multifactorial, not clearly standardized, assessment of continued suitability for endocrine-based treatment [12–14]. In clinical practice, this distinction often reflects a continuum informed by (Figure 1): *i*) urgency for treatment response, including symptomatic or high disease burden and possibly impending organ dysfunction, *ii*) anticipated benefit of another endocrine-based therapy (evaluated by prior treatment history and biomarker profile, amongst others), *iii*) general health condition, comorbidities and preferences of the patient [12–14].

**Figure 2:**
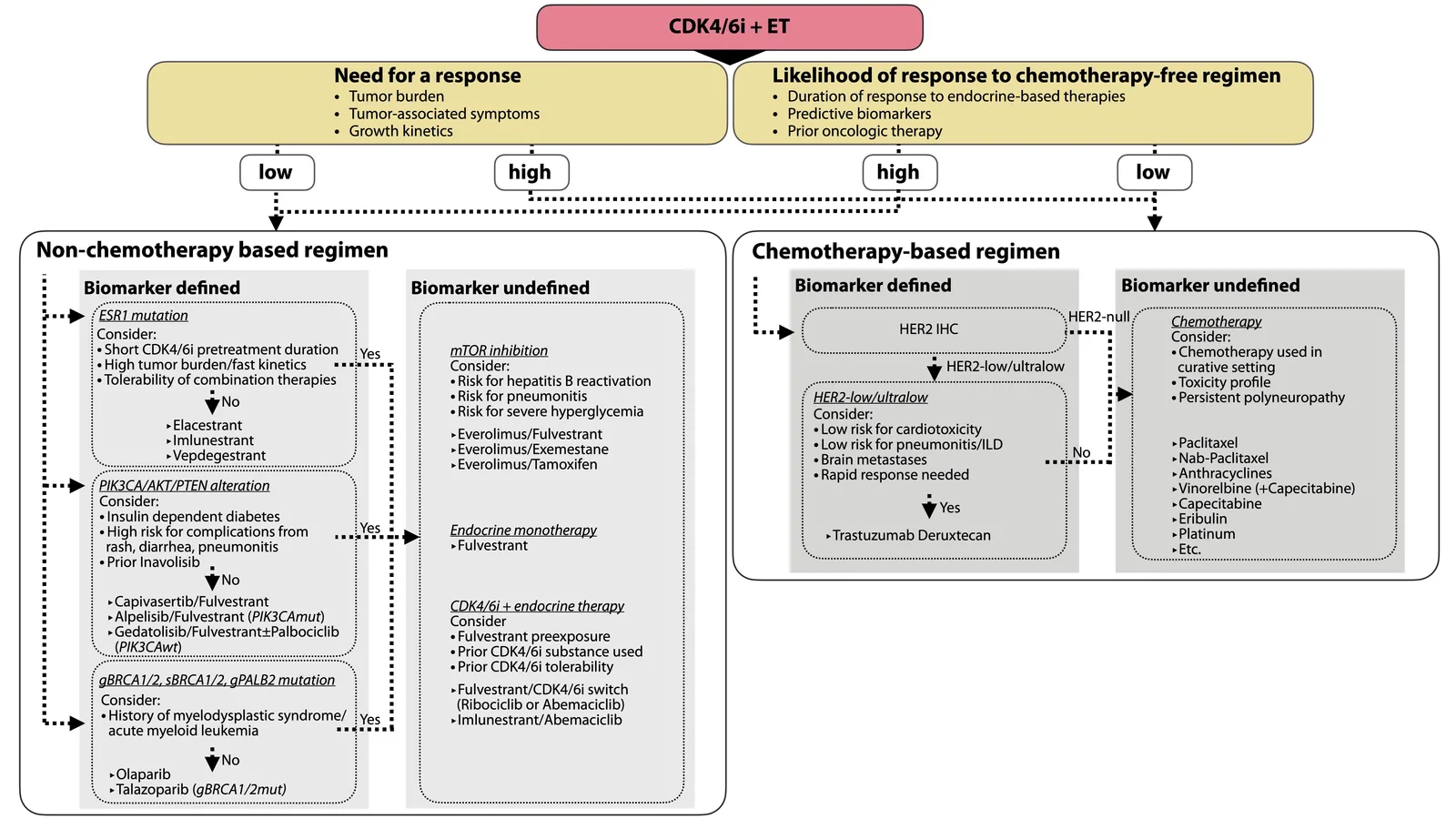
**Potential decision framework in advanced HR+/HER2– breast cancer following CDK4/6i progression.** The proposed algorithm reflects the authors’ opinion. Selection of novel treatment strategies was restricted to options with fully published trial results. HER2-null refers to the complete absence of membranous staining, i.e., HER2– status not meeting the criteria for HER2-low or HER2-ultralow [106]. CDK4/6i: CDK4/6 inhibitor; ET: endocrine therapy; *gBRCA1/2*: germline *BRCA1/2*; *gPALB2*: germline *PALB2*; HER2–: HER2-negative; HR+: hormone receptor-positive; IHC: immunohistochemistry; ILD: interstitial lung disease; mTOR: mechanistic target of rapamycin; s*BRCA1/2*: somatic *BRCA1/2.* Icons provided by Apple Keynote.

In patients still considered candidates for endocrine-based therapy, treatment options are based on switching ET, including novel strategies to target the estrogen receptor (ER), which may be administered 1) as monotherapy, or in combination with 2) CDK4/6i after CDK4/6i, 3) inhibitors of the PI3K/AKT/mTOR pathway, or 4) increasingly investigated, a combination of the aforementioned agents. In patients deemed ineligible for endocrine-based therapy, escalation to chemotherapy or an antibody-drug conjugate (ADC) is warranted (Figure 2). Across these decisions, biomarker profiles are increasingly integrated to guide treatment selection [12–14].

The objective of this narrative review is to provide a structured and comprehensive overview of the most recent randomized clinical trial evidence relevant to guide second-line treatment in HR+/HER2– ABC following progression on CDK4/6i therapy. We aim to address the heterogeneity and limitations of trial designs regarding patient populations, comparator treatment and endpoints, and discuss the resulting constraints in cross-trial interpretation and translation of results into clinical practice. As precision oncology has become a defining feature in this setting, reflected in a one-to-one biomarker-to-treatment matching in both contemporary clinical trial design and clinical practice algorithms, we explore aspects of mechanistic, methodological and clinical uncertainties related to biomarker-guided treatment selection, and discuss aspects of practical guidance within this complexity.

To characterize the current second-line, randomized clinical trial evidence in this narrative review, we conducted a literature search in PubMed using a combination of MeSH terms and free-text terms covering breast cancer, hormone receptor positivity and advanced stage. Publications were restricted to randomized phase II and III trials published between January 2016 and March 10, 2026. Trial evidence from the literature search reporting positive outcomes was included if it aligned with the scope of this review (defined as treatment options post CDK4/6i progression; studies with a more heavily pretreated population were not considered). Negative results were included at the authors’ discretion when they were considered helpful for discussing specific aspects. In addition, an exploratory search of PubMed and Google Scholar was performed, and international oncologic guidelines and resources from major oncology congresses were consulted to identify additional trials and complementary and contextual information. The summary of trial evidence was restricted to results published (in full or abstract form) until March 10, 2026, whereas contextual information was considered until June 30, 2026.

Unless otherwise stated, presented outcomes derive from fully published peer-reviewed clinical trial evidence; where abstract-only data are cited, this is explicitly noted. Subgroup analyses for biomarker-selected patients are reported where they reflect primary or secondary endpoints, or where the trial population was restricted to a biomarker-selected subgroup, unless otherwise stated. Exploratory subgroup analyses stratified by CDK4/6i pretreatment were preferentially reported (and explicitly labeled as such), given their relevance to the scope of the review.

## Endocrine resistance(s)

The term endocrine resistance carries a context-dependent meaning in second-line treatment of advanced, HR+/HER2– breast cancer. A most widely established clinical definition was introduced by the international ABC consensus guidelines, which has since been continuously adapted and which distinguishes between primary and secondary endocrine resistance. Primary endocrine resistance has initially been defined as relapse while on the first 2 years of adjuvant ET or progressive disease within the first 6 months of first-line ET in the advanced setting; whereas secondary endocrine resistance has referred to progression on adjuvant ET after the first two years, relapse within 12 months of completing adjuvant ET, or progressive disease ≥ 6 months on ET for ABC [15]. Those definitions have been updated to include progression on endocrine-based therapies, which also includes CDK4/6i use, and to extend the definition of secondary endocrine resistance to progression on any second-line endocrine-based therapy, and to the presence of *ESR1* mutations [16]. However, the distinction between primary and secondary endocrine resistance as such has not primarily been used to guide clinical decision-making for first-line and second-line therapies in the advanced setting, but rather serves to classify patient populations for clinical trials [16]. The recently published ESMO guidelines on metastatic breast cancer (available as pre-proof) also reference these definitions, but do not integrate them into their treatment recommendations. At the same time, they introduce the term endocrine-sensitive disease, for which they recommend at least two endocrine-based therapy lines, without clear characterization of the term [13]. Similarly, the NCCN Clinical Practice Guidelines in Oncology (NCCN Guidelines^®^) use the term endocrine-refractory disease to guide treatment decisions, without a precise definition of the term and categorization being based on clinical judgement [14].

Mechanistically, endocrine resistance encompasses the cellular and molecular processes by which tumors acquire resistance to ET. Endocrine resistance mechanisms have initially been conceptualized in the context of endocrine monotherapy, involving distinct ways by which tumor cells may either reactivate ER signaling despite ET—most prominently exemplified by *ESR1* mutations—or bypass ER signaling via activation of alternative signaling pathways or changes in transcription factors distinct from ER [17]. With CDK4/6i as a component of first-line and adjuvant therapy, resistance mechanisms under combined ET and CDK4/6i therapy have gained clinical and research interest.

The kinases CDK4 and 6, upon binding cyclin D, phosphorylate the retinoblastoma tumor suppressor protein (Rb), which leads to dissociation of the transcription factor E2F from the Rb/E2F complex and consequently promotes E2F-mediated transcription of genes driving cell cycle progression. CDK4/6i inhibit CDK4 and CDK6 kinase activity and Rb phosphorylation, thereby preventing E2F-driven cell cycle progression. Mechanisms of resistance to CDK4/6i are numerous, such as functional Rb loss, upregulation of the CDK2-Cyclin E complex (and thereby bypassing CDK4/6 inhibition by alternative Rb phosphorylation), alternative native conformational states of CDK4 and 6, and activation of different growth factor pathways, amongst others [17, 18]. While endocrine resistance remains a widely used term in the second-line setting, it nowadays encompasses resistance to both ET and CDK4/6i, refers to a landscape of resistance mechanisms beyond those induced by ET alone, and lacks a clinically actionable, precise definition. In the following, the term endocrine-resistant refers to trial populations with either primary or secondary resistance to ET.

## Current first-line treatment landscape

CDK4/6i have evolved as a cornerstone of palliative therapy of HR+/HER2– breast cancer. The CDK4/6i abemaciclib, palbociclib, and ribociclib have demonstrated significant improvements in progression-free survival (PFS) when combined with ET in endocrine-naïve, endocrine-pretreated but not resistant, and endocrine-resistant advanced disease [19–24]. Abemaciclib added to ET additionally resulted in a clinically meaningful improvement in overall survival (OS), but reached statistical significance only in endocrine-resistant disease [5, 11]. Ribociclib likewise demonstrated an OS benefit in both endocrine-naïve or non-resistant and mixed populations [4, 8]. Based on the OS benefit observed with adjuvant abemaciclib and the invasive disease-free survival (iDFS) benefit reported with adjuvant ribociclib in high-risk early-stage HR+/HER2– breast cancer, CDK4/6i have expanded into the curative setting [3, 10]. Treatment options for first-line palliative therapy in patients relapsing during or after adjuvant CDK4/6i resemble those in patients progressing on CDK4/6i in the metastatic setting. Potential differences in tumor biology and treatment responses between these groups remain insufficiently explored, and evidence to guide therapy selection in the post-adjuvant-CDK4/6i setting is largely missing. In analogy to clinical endocrine resistance mechanisms, suggestions have been made to incorporate definitions of CDK4/6i resistance—including time on or after CDK4/6i prior to recurrence—to inform therapeutic decision-making [25].

The established first-line treatment strategy of ET plus CDK4/6i has been refined following the results of the phase III INAVO120 trial, in which the addition of the PI3K inhibitor inavolisib to palbociclib and fulvestrant improved OS in a *PIK3CA*-mutated, CDK4/6i-naïve population progressing on or shortly after adjuvant ET [26]. Interpretation of these results is complicated by the uncertain significance of an OS benefit compared with palbociclib plus fulvestrant, a regimen which itself did not improve OS in this setting [7]. With the triplet regimen incorporating CDK4/6i and PI3K/AKT/mTOR pathway-targeting agents moving into the first-line setting, evidence to guide subsequent treatment is lacking. Other first-line strategies under investigation include intensified combination approaches and the earlier use of established second-line or novel therapeutic agents: Ongoing phase III trials evaluating CDK4/6i in the first-line setting in endocrine-sensitive disease (generally allowing for adjuvant CDK4/6i exposure) evaluate triplet regimens with aromatase inhibitor (AI) and the PI3K inhibitor inavolisib [27], or doublet combinations with novel endocrine agents [28–30], or even challenge the first-line ET plus CDK4/6i approach with the introduction of the more selective CDK4 inhibitor (CDK4i) atirmociclib [31]. If positive, these trials might change or amend the first-line palliative treatment landscape and will consequently reshape choices for second-line treatment. Additionally, in the phase III lidERA trial, results (presented as an abstract) demonstrated that the oral selective ER degrader (SERD) giredestrant showed a benefit in iDFS compared to standard of care (SOC) ET, possibly generating a role of SERD in earlier lines of treatment [32].

To date, most patients requiring palliative second-line therapy in HR+/HER2– ABC have received ET in combination with a CDK4/6i. In the following, we focus on therapeutic options in this treatment setting, recognizing that further adjustments may be required in the near future.

## Endocrine-based therapies

### Endocrine monotherapies

Inhibition of ER signaling remains the mainstay of palliative therapy in HR+/HER2– breast cancer and can be achieved through estrogen suppression or direct ER targeting, which represents the mechanism of action of several novel agents, including SERDs, proteolysis-targeting chimeras (PROTACs), selective ER modulators (SERMs) and complete ER antagonists (CERANs) [33].

Mutations in the *ESR1* gene encoding the ER are an acquired resistance mechanism to ET. *ESR1* mutations induce conformational changes, constitutive activation, and increased proteolytic stability of the ER, thereby inducing resistance to estrogen-depleting approaches with AI, and to a variable extent, different agents directly targeting the ER [34]. *ESR1* mutations are rare in treatment-naïve HR+/HER2− breast cancer, but occur at a prevalence up to 20–40% in advanced disease treated with AI [34]. They have been shown to arise during AI therapy, and to precede and correlate with clinical progression [35, 36]. In contrast to AI, which reduces ER signaling via estrogen deprivation, SERDs inhibit ER-induced downstream signaling by directly binding to the ER and inducing its proteasomal degradation [37].

Fulvestrant had long been the only SERD available for treatment of HR+/HER2– metastatic breast cancer. Following progression on a CDK4/6i, a review of recent randomized phase II–III trials using fulvestrant as control therapy has shown a median PFS of around 3.2 months (range 1.9–5.3 months), consistent with real-world outcomes [38–40]. Given the limited clinical activity of fulvestrant after CDK4/6i and its intramuscular route of application due to poor oral bioavailability, oral SERDs were developed to improve potency and enable more convenient oral administration [37].

Elacestrant was the first oral SERD to demonstrate efficacy as monotherapy after mandatory prior CDK4/6i exposure and a maximum of one prior line of chemotherapy in a phase III trial. In the EMERALD study, elacestrant was compared with investigator’s choice (IC) of ET (IC ET) (AI or fulvestrant) in patients with disease progression on prior ET plus a CDK4/6i. The trial met its two primary endpoints, demonstrating improved PFS in both the overall population and with greater benefit in the *ESR1*-mutated subgroup (median PFS 3.8 months with elacestrant vs. 1.9 months for IC ET; hazard ratio (HR) 0.55 [95% confidence interval (CI) 0.39–0.77]; *P* = 0.0005). Landmark analyses demonstrated a 6-month PFS rate of 40.8% with elacestrant vs. 19.1% with IC ET in the *ESR1*-mutated subgroup. A secondary analysis excluded the 31% of patients receiving AIs in the control arm, accounting for the recognized resistance associated with *ESR1* mutations. Elacestrant continued to demonstrate benefit over fulvestrant, again with a more pronounced effect in the *ESR1*-mutated cohort [6-month PFS rate 40.8% vs. 20.8%; HR 0.50 (95% CI 0.34–0.74); *P* = 0.005] [41]. The most common adverse events (AEs) (any grade) with elacestrant were nausea (35%), fatigue (19%), vomiting (19%), decreased appetite (14.8%) and arthralgia (14.3%). Among grade ≥ 3 events, nausea (2.5%) and back pain (2.5%) occurred most frequently, followed by increase in alanine aminotransferase (ALT) and aspartate aminotransferase (AST) (2.1% and 1.7%, respectively) and headache (1.7%). Treatment-related AEs led to treatment discontinuation in 3.4% of patients (Table 1) [41]. No OS benefit was shown at the final OS analysis in either the intention-to-treat (ITT) or the *ESR1*-mutated population (both key secondary endpoints; 51.3% of the ITT population experienced an event) [42]. A separately published, post-hoc exploratory analysis by pretreatment duration in exclusively *ESR1*-mutated patients has been considerably discussed in the field. Longer prior CDK4/6i exposure (≥ 12 months) was associated with improved PFS on elacestrant vs. IC ET [8.6 months vs. 1.9 months, HR 0.41 (95% CI 0.26–0.63)] in *ESR1*-mutated patients, compared to a shorter pretreatment period [43].

**Table 1:** Selected novel endocrine agents with proven single-agent efficacy post-CDK4/6i: approval status and AE profiles.

**Substance**	**Indication EMA^1^**	**Indication FDA^1^**	**Incidence of AEs (% of patients)**		**Incidence of treatment-related AEs (% of patients)**					
			**Most common, any grade^2^**	**Most common, ≥ G3^2^**	**Most common, any grade^2^**	**Most common, ≥ G3^2^**	**Overall, any grade**	**Overall, ≥ G3**	**Overall, G5**	**Leading to treatment discontinuation**
Elacestrant	*ESR1*-mutated, after ≥ 1 line of ET including a CDK4/6i [142]	*ESR1*-mutated, after ≥ 1 line of ET [143]	Nausea (35) Fatigue (19) Vomiting (19) Decreased appetite (14.8) Arthralgia (14.3) [41]	Nausea (2.5) Back pain (2.5) ALT increased (2.1) AST increased (1.7) Headache (1.7) [41]	Nausea (25.3) Fatigue (11) Vomiting (11) Hot flush (9.7) Decreased appetite (7.6) Diarrhea (7.6) [41]	Nausea (1.7) Fatigue (0.8) Vomiting (0.4) Decreased appetite (0.4) Abdominal pain (0.4) [41]	63.3 [41]	7.2 [41]	0 [41]	3.4 [41]
Imlunestrant	*ESR1*-mutated, after endocrine-based regimen [144]	*ESR1*-mutated, after ≥ 1 line of ET [145]	Fatigue/Asthenia (22.6) Diarrhea (21.4) Nausea (17.1) Arthralgia (14.1) AST increased (12.5) [46]	Anemia (2.1) Neutropenia (2.1) AST increased (0.9) Thrombopenia (0.9) Arthralgia (0.6) Back pain (0.6) Vomiting (0.6) Leukopenia (0.6) [46]	Not reported [46]	Not reported [46]	48.9 [46]	4.6 [46]	0.3^3^ [46]	2 [47]
Camizestrant (75 mg)	Not approved [88]	Not approved [87]	TEAEs:^4^ Photopsia (12) Anemia (10.8) Cough (9.5) Asthenia (8.1) Nausea (8.1) [44]	No TEAE ≥ G3 occurred in > 1 patient^4, 5^ [44]	Not reported [44]	Not reported [44]	53 [44]	1 [44]	0 [44]	3 [44]
Vepdegestrant	Not approved [88]	*ESR1*-mutated, after ≥ 1 line of ET [146]	Fatigue/Asthenia (26.6) ALT increased (14.4) AST increased (14.4) Nausea (13.5) Anemia (12.2) [52]	Neutropenia (1.9) Hypokalemia (1.9) Anemia (1.6) QT prolonged (1.6) Hypertension (1.6) [52]	Fatigue/Asthenia (18.6) ALT increased (9.6) AST increased (9) Neutropenia (8.7) QT prolonged (8) [52]	Neutropenia (1.9) Fatigue/Asthenia (1) ALT increased (0.6) QT prolonged (0.6) Arthralgia (0.6) [52]	56.7 [52]	7.7 [52]	0 [52]	1.6 [52]

^1^ All indications refer to advanced, HR+/HER2– breast cancer; ^2^ the five most common AEs are listed; ties at 5th rank are all included; ^3^ death due to right ventricular failure; ^4^ for Camizestrant (SERENA-2 trial), incidence of specific AEs and treatment-related AEs were not reported. Therefore, treatment-emergent AEs (TEAEs) are listed instead. Definition of treatment-emergent AE (from SERENA-2 protocol): events will be defined as treatment-emergent if they onset or worsen during the treatment period or safety follow-up period; ^5^ numerous ≥ G3 TEAEs each occurred in only a single patient; these individual events are not listed for clarity. AE: adverse event; ALT: alanine aminotransferase; AST: aspartate aminotransferase; CDK4/6i: CDK4/6 inhibitor; EMA: European Medicines Agency; ET: endocrine therapy; FDA: Food and Drug Administration; G: grade; HER2–: HER2-negative; HR+: hormone receptor-positive.

The oral SERD camizestrant has likewise shown clinical activity in the randomized phase II SERENA-2 trial, in which two dose levels were compared with fulvestrant following disease progression on ET, with optional prior CDK4/6i and chemotherapy exposure for advanced disease [44]. *ESR1* mutations were detected in 48% of patients in the fulvestrant group and in 30% of those receiving the camizestrant dose [44] that was subsequently selected for further evaluation in the SERENA-4 and 6 trials [28, 45]. At this dose level, camizestrant improved PFS compared with fulvestrant [median PFS 7.2 vs. 3.7 months for camizestrant and fulvestrant, respectively; HR 0.59 (90% CI 0.42–0.82); *P* = 0.017]. In the prespecified exploratory analyses by *ESR1* mutation status and prior CDK4/6i exposure (51% of trial population), the relative benefit of camizestrant seemed maintained in patients with *ESR1* mutation [HR 0.33 (90% CI 0.18–0.58)], but not in patients without *ESR1* mutation [HR 0.80 (90% CI 0.51–1.27)]. Treatment effect appeared sustained in patients with prior CDK4/6i exposure [HR 0.49 (90% CI 0.31–0.75)], but not without prior CDK4/6i treatment [HR 0.81 (90% CI 0.49–1.33)]. Most common treatment-emergent AEs (any grade) with camizestrant 75 mg were photopsia (12%), anemia (10.8%), cough (9.5%), asthenia (8.1%), and nausea (8.1%). Treatment-related AEs leading to discontinuation in the camizestrant 75 mg group occurred in 3% of patients. No specific treatment-emergent or treatment-related grade ≥ 3 event was observed in > 1 patient (Table 1). OS data were immature [44]. Camizestrant has been evaluated in combination with a CDK4/6i upon molecular progression in the SERENA-6 trial, as discussed below, and is currently being assessed with palbociclib in the first-line setting in the SERENA-4 trial [28, 45].

The three-arm phase III EMBER-3 trial compared the oral SERD imlunestrant to IC ET (exemestane or fulvestrant) and to imlunestrant plus abemaciclib. Patients were eligible with endocrine-resistant disease after adjuvant or first-line AI therapy, with or without prior CDK4/6i therapy, and no chemotherapy for advanced disease was permitted. Ninety percent of patients in the control arm received fulvestrant, and 10% received exemestane [46]. The trial was negative for its primary endpoint PFS with imlunestrant vs. IC ET in the overall population, but a statistically significant PFS benefit [median PFS 5.5 months with imlunestrant and 3.8 months with IC ET; HR 0.62 (95% CI 0.47*–*0.82); *P* < 0.001] in the *ESR1*-mutated group—another primary endpoint—was demonstrated with initial drop and later separation of the Kaplan–Meier curves [46, 47]. Exploratory subgroup analysis of the *ESR1*-mutated cohort by prior CDK4/6i therapy showed an HR of 0.42 (95% CI 0.25*–*0.72) in CDK4/6i-naïve and 0.72 (95% CI 0.52*–*1.01) in CDK4/6i-pretreated patients (70% of the *ESR1*-mutated population, predominantly palbociclib) [46]. A numerical improvement in OS (key secondary endpoint) was observed in favor of imlunestrant without reaching statistical significance in the *ESR1*-mutated cohort [median OS 34.5 vs. 23.1 months, respectively; HR 0.60 (95% CI 0.43–0.86); *P* = 0.0043, not significant] [47]. Single-agent ET with imlunestrant was inferior to the combination therapy with abemaciclib, as will be outlined below [46, 47]. The most common AEs of any grade were fatigue/asthenia (22.6%), diarrhea (21.4%), nausea (17.1%), arthralgia (14.1%), and AST increase (12.5%). AEs of grade ≥ 3 were anemia and neutropenia (2.1% each), AST increase and thrombopenia (0.9% each) [46]. Treatment-related AEs led to treatment discontinuation in 2% of patients (Table 1) [47].

The randomized trials evaluating the oral SERDs amcenestrant and giredestrant as monotherapy yielded negative results against IC ET in endocrine-resistant populations with varying degrees of prior CDK4/6i exposure [48, 49]. Giredestrant was positive for its primary endpoint, PFS, in the phase III evERA trial in combination with everolimus (full publication results pending), as described below [50], and is currently investigated with a CDK4/6i in phase III trials in the first-line setting in endocrine-sensitive and endocrine-resistant disease [29, 51].

PROTACs represent an additional class of novel endocrine agents with proven clinical efficacy in a phase III trial [52]. PROTACs act by recruiting a ubiquitin ligase to a target protein, promoting its polyubiquitination and subsequent proteasomal degradation [33]. Vepdegestrant, a first-in-class PROTAC targeting the ER [33], demonstrated superiority over fulvestrant in the phase III VERITAC-2 trial in an endocrine-resistant, post-CDK4/6i population [52]. Patients had to be on their most recent ET for ≥ 6 months before progression, and no prior chemotherapy or fulvestrant for advanced disease was allowed. Vepdegestrant did not meet the primary endpoint of PFS in the ITT population but demonstrated superiority in the primary endpoint of PFS in the *ESR1*-mutated subgroup [median PFS 5.0 vs. 2.1 months for vepdegestrant and fulvestrant, respectively; HR 0.58 (95% CI 0.43–0.78); *P* < 0.001]. Data on OS were immature. Most common AEs were fatigue/asthenia (26.6%), ALT or AST increased (14.4% each), nausea (13.5%), and anemia (12.2%). The most common AE grade ≥ 3 were neutropenia, hypokalemia (1.9% each), anemia, QT prolongation, and hypertension (1.6% each, Table 1) [52].

Lasofoxifene is a next-generation SERM and has been tested against fulvestrant in the phase II ELAINE-1 trial in an exclusively *ESR1*-mutated cohort progressing on AI and CDK4/6i therapy. The trial was negative for its primary endpoint, PFS [median 5.6 months for lasofoxifene and 3.4 months for fulvestrant; HR 0.70 (95% CI 0.43–1.15); *P* = 0.14] [53].

CERANs are another novel class of drugs targeting the ER and function as complete estrogen antagonists by blocking the ligand-binding pocket of the ER. Palazestrant is currently being evaluated in the phase III OPERA-01 trial in endocrine-resistant, CDK4/6i pretreated patients against endocrine monotherapy [33, 54].

In conclusion, the three endocrine agents elacestrant, imlunestrant, and vepdegestrant have each demonstrated efficacy as monotherapy in randomized phase III trials; with an *ESR1*-mutated subgroup included as the primary analysis population. While elacestrant showed a PFS benefit across a biomarker-unselected and the *ESR1*-mutated population, the benefit with imlunestrant and vepdegestrant was confined to the *ESR1*-mutated subgroup [41, 46, 52]. These findings should be interpreted in the context of some methodological considerations regarding trial design and cross-trial heterogeneity complicating treatment comparisons (Table 2). All three trials employed ET monotherapy as control treatment, which may not reflect current clinical practice where combination approaches are increasingly preferred. Comparator selection differed across trials, which is a relevant aspect as suboptimal treatment in the control arm may inflate observed treatment effects. While prior fulvestrant was an exclusion criterion in both the vepdegestrant and imlunestrant trials—consistent with fulvestrant being the control treatment—a minority of patients in the imlunestrant trials received AI therapy as control treatment despite mandatory AI preexposure; and in the elacestrant trial, both fulvestrant and AI were used as control treatments despite prior exposure being allowed. In addition, CDK4/6i pretreatment was mandatory only in the vepdegestrant and elacestrant trials, limiting applicability of the imlunestrant results to a post-CDK4/6i setting [41, 46, 52]. Moreover, the included population was heavily pretreated in the elacestrant trial, which may have attenuated the observed treatment effect, as treatment efficacy generally decreases with increasing lines of systemic therapy [41]. Across the three trials, patients with indeterminate *ESR1* mutational status were classified and analyzed as *ESR1*-non-detectable. However, reporting of the prevalence of *ESR1*-negative and *ESR1-*indeterminate within the *ESR1*-non-detectable group was inconsistent between trials, and *ESR1* mutational status was not a stratification factor in all three trials (Table 3) [41, 46, 52]. Camizestrant as monotherapy only demonstrated activity in a phase II setting, with the clinically relevant subgroup of patients with CDK4/6i preexposure limited to fewer than 40 patients per arm; the clinical significance of these findings therefore remains to be confirmed in a larger trial [44].

**Table 2:** Overview of positive phase II and III randomized clinical trials with ET-based therapy following progression on CDK4/6i.

**Trial name (intervention, if multiple arms)**	**Phase**	**Primary endpoint(s)**	**Biomarker (% of patients with biomarker)**	**Pretreatment history (% of patients)**				**Intervention**	**Control**	**PFS in ITT [HR (95% CI)^2^,** ***P*-value]**	**PFS in biomarker-defined group^1^ [HR (95% CI)^2^,** ***P*-value)]**
				**≥ 2 ETs^3^**	**CDK4/6i**	**ChT^3^**	**Fulv**				
EMERALD [41]	III	PFS in ITT, PFS in *ESR1*m	*ESR1*m (48)	43.4	Mand.	22.2	30.4	Elacestrant	Fulvestrant/AI	0.70 (0.55−0.88), *P* = 0.002	0.55 (0.39−0.77), *P* < 0.001
SERENA-2 [44] (Camizestrant 75 mg)	II	PFS for each Camizestrant dose level	NA	Excl.	51	23.8	Excl.	Camizestrant (75 mg)	Fulvestrant	0.59 (0.42−0.82)^4^, *P* = 0.017	NA
EMBER-3 [46, 47] (Imlunestrant vs. IC ET)	III	PFS in ITT; PFS in *ESR1*m	*ESR1*m (38.7)	Excl.	58.1	Excl.	Excl.	Imlunestrant	Fulvestrant/AI	0.89 (0.75−1.05), *P* = 0.167	0.62 (0.47−0.82), *P* < 0.001
EMBER-3 [46, 47] (Imlunestrant, Abemaciclib vs. Imlunestrant)	III	PFS in ITT	NA	Excl.	65.5	Excl.	Excl.	Imlunestrant Abemaciclib	Imlunestrant	0.59 (0.47−0.74), *P* < 0.001	NA
VERITAC-2 [52]	III	PFS in ITT, PFS in *ESR1*m	*ESR1*m (43.3)	NR^5^	Mand.	Excl.	Excl.	Vepdegestrant	Fulvestrant	0.83 (0.69−1.01), *P* = 0.07	0.58 (0.43−0.78), *P* < 0.001
MAINTAIN [59]	II	PFS in ITT	NA	18.5	Mand.	9.2	NR	Switched ET (Fulvestrant/AI) Ribociclib	Switched ET (Fulvestrant/AI) Placebo	0.57 (0.39−0.85), *P* = 0.006	NA
postMONARCH [60]	III	PFS in ITT	NA	Excl.	Mand.	Excl.	Excl.	Fulvestrant Abemaciclib	Fulvestrant Placebo	0.73 (0.57−0.95), *P* = 0.017	NA
CAPItello-291 [82]	III	PFS in ITT, PFS in AKT pathway-altered^6^	AKT pathway-altered^6^ (40.8)	10.7	69.1	18.2	Excl.	Fulvestrant Capivasertib	Fulvestrant Placebo	0.60 (0.51−0.71), *P* < 0.001	0.50 (0.38−0.65), *P* < 0.001
VIKTORIA-1 [86] (Fulvestrant, Gedatolisib, Palbociclib vs. Fulvestrant)	III	PFS in *PIK3CA*-WT = ITT	*PIK3CA*-WT (100)	10.3	Mand.	Excl.	NR^5^	Fulvestrant Gedatolisib Palbociclib	Fulvestrant	0.24 (0.17−0.35), *P* < 0.001	Identical to ITT
VIKTORIA-1 [86] (Gedatolisib, Fulvestrant vs. Fulvestrant)	III	PFS in *PIK3CA*-WT = ITT	*PIK3CA-*WT (100)	10.3	Mand.	Excl.	NR^5^	Fulvestrant Gedatolisib	Fulvestrant	0.33 (0.24−0.48), *P* < 0.001	Identical to ITT
SERENA-6 [45]	III	PFS in *ESR1*m = ITT	*ESR1*m (100)	Excl.	Mand.	5.4	Excl.	Camizestrant Palbociclib/Ribociclib or Abemaciclib	AI Palbociclib/Ribociclib or Abemaciclib	0.44 (0.31−0.60), *P* < 0.001	Identical to ITT
PADA-1 [57]	III	PFS in *ESR1*m = ITT	*ESR1*m (100)	Excl.	Mand.	Excl.	Excl.	Fulvestrant Palbociclib	AI Palbociclib	0.61 (0.43–0.86), *P* = 0.004	Identical to ITT

Only trials with full publication are included. ^1^ Only reported where efficacy in biomarker-defined population was a primary endpoint; ^2^ unless otherwise specified; ^3^ in the advanced setting; ^4^ 90% CI; ^5^ inclusion possible; ^6^ molecular alterations in *PIK3CA*, *AKT1*, or *PTEN.* AI: aromatase inhibitor; CDK4/6i: CDK4/6 inhibitor; ChT: chemotherapy; CI: confidence interval; *ESR1*m: *ESR1*-mutated; ET: endocrine therapy; Excl.: excluded; Fulv: fulvestrant; HR: hazard ratio; ITT: intention-to-treat; Mand.: mandatory; NA: not applicable; NR: not reported; PFS: progression-free survival; IC ET: investigator's choice of ET; WT: wild-type.

**Table 3:** Biomarker testing in positive, randomized phase III trials in the post-CDK4/6i setting in advanced, HR+/HER2– breast cancer.

**Trial**	**Biomarker**	**Specimen source and timing for biomarker testing**	**Biomarker assessment site**	**Stratified according to biomarker**	**Assay**	**Included alterations**	**Rule for assignment of biomarker-indeterminate patients**	**Patient distribution according to biomarker status (% of ITT)**
VIKTORIA-1 [86]	*PIK3CA*-WT	Archival or fresh biopsy. If neither available: ctDNA from blood samples	Central	NA^1^	Therascreen PIK3CA Rotor-Gene Q PCR test (QIAGEN)	10 *PIK3CA* mutations: C420R, E542K, E545A, E545D [1635G>T only], E545G, E545K, Q546E, H1047L, H1047R, H1047Y	Identified *PIK3CA* status was an inclusion criterion	Identified *PIK3CA* status was an inclusion criterion
CAPItello-291 [82]	*PIK3CA*, *AKT1*, *PTEN*	Tumor tissue from the most recently collected tumor sample (primary or recurrent)	Central	No	China: OncoScreen Plus (Burning Rock Biotech) All other countries: FoundationOne CDx (Foundation Medicine)	Activating mutations in *PIK3CA* and *AKT1* and inactivating alterations in *PTEN* genes	Patients without a qualifying alteration or with an unknown test result were included in the AKT pathway-non-altered population	40.8% with confirmed AKT pathway alterations 59.2% without confirmed AKT pathway alterations (of which 25.3% had an unknown AKT pathway alteration status)
VERITAC-2 [52]	*ESR1*	ctDNA from pretreatment blood samples (prior to randomization)	Central (for some patients: local laboratory results were used)	Yes	China: NGS by Origmed All other countries: NGS by Foundation Medicine	Not reported	Patients with non-informative *ESR1* test (result “unknown”) were stratified as patients without *ESR1* mutation	43.3% with *ESR1* mutation 56.7% without *ESR1* mutation (percentage of patients with a possibly unknown result not reported)
EMERALD [41]	*ESR1*	ctDNA in blood samples at screening	Central	Yes	Guardant360 CDx (Guardant Health)	*ESR1* missense mutations in codons 310–547	Patients without detectable ctDNA in the blood sample/where *ESR1* status could not be determined are included in the group of patients without *ESR1* mutation	47.8% with *ESR1* mutation 52.2% without *ESR1* mutation (percentage of patients without detectable ctDNA not reported)
EMBER-3 [46] (Imlunestrant vs. Fulvestrant)	*ESR1*	ctDNA in blood samples before treatment administration (after randomization)	Central	No	China: OncoCompass Target (Burning Rock Biotech) All other countries: Guardant360 CDx (Guardant Health)	34 *ESR1* variants annotated as oncogenic or likely oncogenic by the OncoKB database	Patients without detectable ctDNA or with an unknown *ESR1* mutation status (analytical failure, missing sample) are included in the group of patients without *ESR1* mutation	38.7% with *ESR1* mutation 58.2% without *ESR1* mutation (of which 5% have an unknown *ESR1* mutation status)
PADA-1 [57, 58]	*ESR1*	ctDNA in blood samples at inclusion and repeatedly (during surveillance^2^ period: every 2 cycles)	Central	NA^1^	Multiplex ddPCR (QX200 system; Bio-Rad Laboratories, Marnes-la-Coquette, France)	*ESR1* mutations in hotspot codons 380, 536, 537, and 538	Rising *ESR1* mutation was an inclusion criterion	Rising *ESR1* mutation was an inclusion criterion After randomization^3^: 46.6% of patients had no *ESR1* mutation detected in pretreatment sample
SERENA-6 [45]	*ESR1*	ctDNA in blood samples at inclusion and repeatedly (during surveillance^2^ period: every 2–3 months)	Central	NA^1^	Guardant360 CDx (Guardant Health)	11 *ESR1* mutations E380Q, V422del, S463P, L536H, L536P, L536R, Y537C, Y537D, Y537N, Y537S, D538G	*ESR1* mutation was an inclusion criterion	*ESR1* mutation was an inclusion criterion
DESTINY-Breast06 [97]	HER2-low HER2-ultralow	Tissue sample at time of metastatic disease or later (most recent pre-randomization tumor sample)	Central	Yes (HER2-low vs. ultralow)	VENTANA HER2/neu (4B5) assay	HER2-low: IHC 1+ or IHC 2+ and ISH− HER2-ultralow: IHC 0 with membrane staining; also known as IHC > 0 and < 1+	HER2-low or HER2-ultralow status was an inclusion criterion	81.8% HER2-low 17.6% HER2-ultralow

Biomarker testing strategies and ascertainment rules in randomized phase III trials in the post-CDK4/6i setting. Trials are presented in which a primary endpoint was evaluated in a prospectively defined biomarker-selected population. Only trials reported as full publications were included. Data presented in this table were derived exclusively from the original or follow-up study report or protocol, no additional sources were consulted. ^1^ Only biomarker-defined patients were included in the study; ^2^ during the surveillance period, patients were monitored for *ESR1*. In case of rising (PADA-1) or detection (SERENA-6) of *ESR1* mutation, patients were randomized to the respective treatments; ^3^ after randomization, *ESR1* testing was repeated before treatment initiation, referred to as the pretreatment sample. CDK4/6i: CDK4/6 inhibitor; ctDNA: circulating tumor DNA; ddPCR: droplet digital polymerase chain reaction; IHC: immunohistochemistry; ISH: in situ hybridization; ITT: intention-to-treat; NA: not applicable; NGS: next-generation sequencing; WT: wild-type.

### CDK4/6i post CDK4/6i-progression: identical CDK4/6i, switch of ET

Given the modest efficacy of endocrine monotherapy following progression on ET and CDK4/6i, the selection of a combination therapy may be favored as a further line therapy. A biomarker-independent strategy consists of either switching to a different endocrine agent and a different CDK4/6i, or switching to an alternative ET while maintaining the same CDK4/6i beyond progression on both agents.

The phase II PALMIRA trial evaluated the continuation of palbociclib beyond disease progression by randomizing patients previously treated with palbociclib plus an AI or fulvestrant to receive either ET alone or ET with continued palbociclib. The trial did not meet the primary endpoint of PFS [55]. Similarly, the phase II PACE trial randomized patients progressing on an AI plus CDK4/6i therapy (palbociclib in 91% of patients) to fulvestrant alone or fulvestrant plus palbociclib, but did not demonstrate a PFS benefit for the combination therapy [56].

Two extensively debated trials introduced a novel treatment approach, in which therapy was not switched upon clinically evident progression, but upon emergence of a molecular marker of progression [45, 57]. Similar to PALMIRA and PACE, the PADA-1 trial evaluated an ET switch with continued CDK4/6 inhibition. Notably, the treatment was changed at the time of molecular progression—defined by rising *ESR1* mutations in circulating tumor DNA (ctDNA)—rather than at clinical progression on first-line AI and palbociclib. Patients with rising *ESR1* mutations were randomized to continue AI plus palbociclib or switch to fulvestrant plus palbociclib. This strategy was associated with a significant improvement in the primary endpoint PFS in favor of the molecularly triggered treatment switch [updated analysis: median PFS 12.8 months and 5.9 months for switch to fulvestrant and continuation of AI, respectively; HR 0.54 (95% CI 0.38–0.75); *P* = 0.0003] [57, 58]. Sixty-eight percent of patients in the control arm crossed over to fulvestrant plus palbociclib after clinically evident progression, with a median PFS of 3.5 months in this subsequent line. The secondary endpoint median time to strategy failure, i.e., time from randomization to time of palbociclib and ET discontinuation or death, was 11.9 months for the switch-fulvestrant-group and 10.6 months for the AI-continuation group [HR 1.02 (95% CI 0.71–1.45); *P* = 0.9; from primary analysis]. Similarly, the median chemotherapy-free survival was 14.6 months in the fulvestrant-switch group compared with 13.1 months in the AI-continuation group [HR 0.91 (95% CI 0.62–1.33); secondary endpoint, *P* = 0.6, from primary analysis] [57]. OS data were still immature [58].

Analogous to PADA-1, SERENA-6 investigated a treatment switch triggered by the detection of *ESR1* mutations by ctDNA prior to clinically evident progression*.* Patients on first-line AI plus CDK4/6i therapy (palbociclib in 76% of patients) were randomized upon *ESR1* mutation detection to either continue the same regimen or to switch the endocrine backbone to the oral SERD camizestrant, while maintaining the same CDK4/6i. Crossover to an oral SERD with CDK4/6i continuation was not allowed in the control arm. In patients with a detectable *ESR1* mutation who were randomized to the respective treatments, PFS was significantly prolonged with camizestrant and CDK4/6i in comparison to continuation of the initial regimen [median PFS 16 months vs. 9.2 months; HR 0.44 (95% CI 0.31–0.60); *P* < 0.0001]. Results for PFS2, which in principle compares an unequal number of sequentially administered therapeutic substances between groups, were immature at first analysis [HR 0.52 (95% CI 0.33–0.81); *P* = 0.004, prespecified significance threshold *P* = 0.0001]. OS data were immature [45].

The PADA-1 and SERENA-6 trials were notable for the pioneering approach to guide treatment decisions based on detection of molecularly defined progression. Beyond the aspiration to improve survival outcomes, potential advantages of a molecularly guided switching approach may theoretically include the prevention of tumor-progression-related complications, symptoms and clinical deterioration, and possible reduction of clinical and financial toxicity from an increasingly ineffective treatment. Despite both trials being positive for their primary endpoint, PFS, this endpoint design does not allow for a conclusion whether switching upon molecular instead of clinical progression is superior in efficacy, given the inherent imbalance of active substances between treatment arms. In contrast, the lack of differences in median time to strategy failure and chemotherapy-free survival between the two arms in PADA-1 does not appear to indicate a benefit of therapy adaptation upon molecular rather than clinically evident progression. Furthermore, the definition of molecular progression represents a conceptual challenge. Longitudinal ctDNA analyses from PADA-1 suggest that *ESR1* mutation detectability is dynamic, with fluctuations and occasional loss of detection occurring independently of treatment changes, including in the AI plus CDK4/6i arm [58]. This might challenge the classification of *ESR1* positivity, its direct attribution as a biomarker of relevant molecular progression, and underscores the need for standardized assays and uniform definitions to reduce risk of possible overtreatment. In addition, the feasibility of regular ctDNA monitoring needs to be determined across all healthcare settings, and associated surveillance demands might carry implications for patient experience and health systems.

In summary, switching the endocrine backbone to fulvestrant while maintaining the same CDK4/6i upon clinical progression has only been investigated for palbociclib and did not improve efficacy [55, 56]. Molecularly guided switching strategies of the endocrine agent on first-line AI and CDK4/6i represent a novel approach with potentially beneficial implications, yet convincing evidence of superiority in efficacy is currently lacking, and questions regarding practical implementation and implications for patients and health system resources remain open [45, 57, 58].

### CDK4/6i post CDK4/6i-progression: switch of CDK4/6i and switch of ET

In the phase II MAINTAIN trial, patients progressing on ET and CDK4/6i (palbociclib in 87% of patients) in the advanced setting underwent a switch to an alternative ET (fulvestrant or AI), either as monotherapy or in combination with ribociclib. Switching ET plus ribociclib led to improved PFS compared to switching to an alternative ET monotherapy [median PFS 5.3 vs. 2.8 months; HR 0.57 (95% CI 0.39–0.85); *P* = 0.006]. No OS analysis was reported [59].

Similarly, in the phase III postMONARCH trial, patients progressing on AI plus CDK4/6i therapy were switched to fulvestrant monotherapy or fulvestrant in combination with abemaciclib. Palbociclib was the most common prior CDK4/6i (59% of patients), followed by ribociclib (33%) and abemaciclib (8%). The addition of abemaciclib led to a statistically significant PFS benefit [median PFS 6.0 vs. 5.3 months, and 6-month PFS rates 50% vs. 37% for the combination therapy and fulvestrant, respectively; HR 0.73 (95% CI 0.57–0.95); *P* = 0.017]. Exploratory subgroup analyses suggested a potential benefit among patients with prior palbociclib exposure [HR 0.62 (95% CI 0.44–0.86)], while no effect was observed in the small subgroup of ribociclib-pretreated patients [HR 1.01 (95% CI 0.67–1.51)]. However, the interaction *p*-value was not significant. OS data were immature [60].

The approach of simultaneous endocrine and CDK4/6i switch has been modified to the use of oral SERDs instead of fulvestrant for potentially better efficacy and a more convenient oral formulation. In the three-arm phase III EMBER-3 trial, imlunestrant plus abemaciclib was superior to imlunestrant monotherapy after progression on an AI, which had been combined with a CDK4/6i in 65% of patients (palbociclib in 63%, ribociclib in 27%, and abemaciclib in 8% of cases) [47]. The combination of imlunestrant and abemaciclib was superior to imlunestrant alone with respect to the primary endpoint PFS in the ITT population [median PFS 10.9 vs. 5.5 months; HR 0.59 (95% CI 0.47–0.74); *P* < 0.001] [47]. A benefit for the combination therapy was observed in a prespecified exploratory analysis both among patients with prior CDK4/6i treatment [median PFS 9.1 vs. 3.7 months; HR 0.53 (95% CI 0.40–0.69)] and across *ESR1*-mutated and non-mutated subgroups [46, 47]. OS data were not mature yet [47].

In the single-arm phase II ELAINE-2 trial, which exclusively enrolled patients with *ESR1*-mutated disease, lasofoxifene plus abemaciclib demonstrated encouraging activity (median PFS 13 months and overall response rate (ORR) 56%) in a more heavily pretreated population allowing previous ET, chemotherapy, and multiple CDK4/6i exposures [61]. This combination will be compared against abemaciclib and fulvestrant in the phase III ELAINE-3 trial in *ESR1*-mutated disease following progression on CDK4/6i and AI, addressing the need for head-to-head comparisons of combination therapies [62].

Taken together, after progression on ET plus CDK4/6i, switching ET and adding abemaciclib or ribociclib improved PFS vs. endocrine monotherapy [47, 59, 60]. However, this benefit has mainly been shown after prior palbociclib, and its efficacy following abemaciclib or ribociclib remains less certain [47, 59, 60]. The approach of combining imlunestrant with abemaciclib demonstrated a median PFS of 10.9 months, which appears to be an encouraging result. However, the notable limitation of only 61% CDK4/6i pretreatment complicates comparison with other regimens and limits applicability to the post-CDK4/6i setting [47].

### Therapeutic combinations targeting the PI3K/AKT/mTOR signaling pathway

The PI3K/AKT/mTOR signaling pathway plays an important role in cell proliferation, growth, survival, and metabolism [63]. Intracellular activation of the lipid kinase PI3K leads to recruitment of the serine/threonine kinase AKT, which is further activated through phosphorylation by mTORC2. mTORC1 and 2 represent two complexes with distinct functions that both incorporate the serine/threonine kinase mTOR. Activated AKT is a central signaling node of the pathway and mediates further downstream signaling on multiple target proteins, including activation of mTORC1 among downstream consequences. In contrast, the phosphatase PTEN inhibits PI3K/AKT signaling by antagonizing AKT activation [63, 64].

In HR+/HER2– ABC, the mTOR inhibitor everolimus was the first clinically introduced substance to interfere with the PI3K/AKT/mTOR pathway and inhibits mTORC1 [64, 65]. Everolimus showed therapeutic efficacy in clinical trials in an endocrine-resistant population in combination with ET in the pre-CDK4/6i-era [66–68]. Treatment with everolimus has not been biomarker-guided, and an association with treatment efficacy with *PIK3CA* mutation has not been established [69, 70].

More recently, approaches targeting the PI3K/AKT/mTOR pathway in combination with fulvestrant in a biomarker-selected population have been implemented in the treatment landscape of HR+/HER2– ABC: In contrast to *ESR1* mutations acquired during treatment, *PIK3CA* mutations—encoding the catalytic subunit of PI3K (p110α)—are mostly considered early, tumor-intrinsic (“truncal”) events and have been observed in up to approximately 35–45% of HR+/HER2– breast cancers, although there is evidence demonstrating spatial and temporal heterogeneity [71–75].

The PI3K inhibitor alpelisib was the first PI3K inhibitor implemented in the treatment of HR+/HER2– ABC [76]. Alpelisib targets the p110α subunit of PI3K [64]. In endocrine-pretreated patients, alpelisib plus fulvestrant demonstrated superiority over fulvestrant for the primary endpoint PFS in a *PIK3CA*-mutated population [median PFS 11 vs. 5.7 months for the combination therapy vs. fulvestrant, respectively; HR 0.65 (95% CI 0.50–0.85); *P* < 0.001] in the phase III SOLAR-1 trial. For the secondary endpoint of PFS in the *PIK3CA* wild-type (WT) patients, no significant effect could be demonstrated [76]. No significant OS benefit was observed [77]. Given that only 6% of patients had received prior CDK4/6i therapy, this treatment was further evaluated in the single-arm BYLieve trial requiring prior CDK4/6i exposure, confirming efficacy in this population (median PFS 8.0 months) [76, 78]. However, alpelisib was associated with relevant toxicity, with a treatment discontinuation rate due to AEs of 26%. The most common grade 3–4 AEs were hyperglycemia (37%), rash (10%), and diarrhea (7%) [77]. Results of the phase III EPIK-B5 trial, available in abstract form only, provided evidence of a significant PFS benefit (primary endpoint) of alpelisib when added to fulvestrant in a post-CDK4/6i and AI setting in an exclusively *PIK3CA*-mutated population [median PFS 7.4 vs. 2.8 months; HR 0.52 (95% CI 0.37–0.72); *P* < 0.001]. The authors stated a positive trend for OS, with a median OS of 29.5 vs. 23.8 months in the control arm [HR 0.64 (95% CI 0.41–0.99); *P* = 0.021]. Statistical significance was not explicitly claimed, and formal interpretation of this *P*-value awaits full publication [79].

Inavolisib, a selective p110α inhibitor that also degrades mutant p110α, has been incorporated into first-line therapy for HR+/HER2– breast cancer in combination with palbociclib and fulvestrant in patients with a *PIK3CA* mutation [26]. Inavolisib plus fulvestrant is being evaluated vs. alpelisib plus fulvestrant in patients with *PIK3CA*-mutated disease after CDK4/6i therapy in the phase III INAVO121 trial, thereby generating important clinical evidence for direct comparisons between endocrine combination therapies [80].

The novel, allosteric PI3Kα inhibitor zovegalisib is designed to target mutant PI3Kα while sparing the WT variant, with the aim of reducing the side effects of PI3Kα-inhibitors [64]. In the phase III ReDiscover-2 trial, zovegalisib plus fulvestrant is being tested against capivasertib and fulvestrant in a CDK4/6i pretreated population with *PIK3CA* mutation, providing important direct comparisons of combination therapies [81].

Apart from mutations in *PIK3CA,* the PI3K/AKT/mTOR pathway can be activated by less prevalent mutations in *AKT1* (5–7% in ABC) and by loss of PTEN function (5–10% of breast cancers), both resulting in AKT activation [64]. The selective AKT inhibitor capivasertib targets PI3K/AKT/mTOR pathway activation driven by genomic alterations in *PIK3CA*, *AKT1*, or *PTEN.* In combination with fulvestrant, capivasertib demonstrated superiority over fulvestrant in the randomized phase III CAPItello-291 trial. Eligible patients had endocrine-resistant advanced HR+/HER2– breast cancer; 70% had prior exposure to CDK4/6i and 18% had received chemotherapy for advanced disease. Forty-one percent of patients had a confirmed PI3K-AKT pathway alteration, with activating *PIK3CA* mutations in 31%, activating *AKT1* mutations in 5%, and inactivating *PTEN* alterations in 5% of patients. Of note, PI3K-AKT pathway alteration status was not assessed before inclusion and not used as a stratification factor at randomization; 15% of patients enrolled had an unknown alteration status (Table 3). Capivasertib plus fulvestrant significantly improved PFS for both primary endpoints: in the ITT population [median PFS 7.2 vs. 3.6 months; HR 0.60 (95% CI 0.51–0.71); *P* < 0.001] and in patients with a PI3K-AKT pathway alteration [median PFS 7.3 vs. 3.1 months; HR 0.50 (95% CI 0.38–0.65); *P* < 0.001]. Exploratory subgroup analysis suggested a benefit irrespective of CDK4/6i pretreatment in the ITT population. An exploratory analysis in the PI3K-AKT pathway-non-altered population, performed after exclusion of the 25% of patients with unknown molecular status, suggested no clear PFS benefit in this subgroup [HR 0.79 (95% CI 0.61–1.02)]. The most common grade ≥ 3 AEs were rash (12%), followed by diarrhea (9%) and hyperglycemia (2.3%). Treatment discontinuation due to AEs occurred in 13% of patients [82]. Capivasertib in combination with fulvestrant and a CDK4/6i is investigated against fulvestrant and CDK4/6i in patients experiencing endocrine-resistant disease in the adjuvant setting in the phase III CAPItello-292 trial [83].

Evidence on another AKT inhibitor, ipatasertib, is currently available as an abstract only, and demonstrated a significant PFS benefit in combination with fulvestrant vs. fulvestrant alone in the phase III CCTG/BCT MA.40/FINER trial. Eligible patients had advanced HR+/HER2– breast cancer progressing on AI and CDK4/6i. The trial was positive for PFS in the ITT population [primary analysis, median PFS 5.3 vs. 1.9 months; HR 0.61 (95% CI 0.46–0.81); *P* = 0.0007], as well as in the PI3K-AKT pathway-altered cohort (secondary analysis), i.e., with genomic alterations in *PIK3CA*, *AKT1,* or *PTEN* [median PFS 5.5 vs. 1.9 months; HR 0.47 (95% CI 0.31–0.72); *P* = 0.0005]. The most common non-hematological grade ≥ 3 AEs were diarrhea (16%), fatigue (3%), vomiting, and rash (2% each). Treatment discontinuation rate due to AEs was 6.5% [84].

In contrast to the previously discussed trials evaluating PI3K and AKT inhibitors with fulvestrant as ET backbone [79, 82, 84], the mTOR inhibitor everolimus has been investigated in combination with the oral SERD giredestrant in the phase III evERA trial [50]. Patients were included upon progression following ET and CDK4/6i and were randomized to either giredestrant plus everolimus or IC ET (i.e., exemestane, fulvestrant or tamoxifen) plus everolimus. The trial was positive for both primary endpoints PFS in the ITT [median PFS 8.8 vs. 5.5 months; HR 0.56 (95% CI 0.44–0.71); *P* < 0.0001] and in the *ESR1-*mutated cohort [median PFS 10 vs. 5.5 months; HR 0.38 (95% CI 0.27–0.54); *P* < 0.0001, presented as abstract] [50]. Full trial publication, along with its clinical positioning within the treatment landscape, remains pending. A clear strength of the trial was the incorporation of a combination-therapy regimen as comparator treatment [50]. Everolimus in combination with the oral SERD elacestrant vs. elacestrant monotherapy is currently investigated in the randomized phase III ADELA trial in the post-CDK4/6i setting in an exclusively *ESR1*-mutated population [85].

Similar to the triplet combination strategy of ET, CDK4/6i and PI3K/AKT/mTOR pathway targeting evaluated in the first-line INAVO120 trial [26], the phase III VIKTORIA-1 trial investigated gedatolisib, palbociclib, and fulvestrant in CDK4/6i pretreated patients [86]. Gedatolisib is a multitarget inhibitor targeting both PI3K and mTORC1/2. The two-part, three-arm trial investigated both *i)* the triplet combination gedatolisib, palbociclib, and fulvestrant and the doublet *ii)* gedatolisib and fulvestrant vs. *iii)* fulvestrant monotherapy. No prior exposure to chemotherapy or ADCs in the advanced setting was allowed. Fulvestrant pretreatment was allowed, but the number of fulvestrant-pretreated patients was not reported. Forty percent of patients had prior palbociclib exposure; 49% ribociclib and 17% prior abemaciclib. Parts 1 and 2 of the study enrolled patients with *PIK3CA*-WT and *PIK3CA*-mutated tumors, respectively. In part 1, PFS was significantly prolonged with the triplet therapy compared to fulvestrant [median PFS 9.3 vs. 2.0 months; HR 0.24 (95% CI 0.17–0.35); *P* < 0.001] and with the doublet therapy compared to fulvestrant [median PFS 7.4 vs. 2.0 months; HR 0.33 (95% CI 0.24–0.48); *P* < 0.001]. The most common grade ≥ 3 treatment-related AEs were neutropenia (62.3% and 0.8% in the triplet and doublet combination group, respectively), stomatitis (19.2% and 12.3%), leukopenia (6.2% and 0%), rash (4.6% and 5.4%), nausea (3.8% and 0.8%), hyperglycemia (2.3% for both groups) and diarrhea (1.5% and 0.8%). Treatment-related AEs led to treatment discontinuation in 2.3% of patients in the triplet combination group and 3.1% in the doublet combination group. Two treatment-related deaths occurred with the triplet combination (pneumonia and hepatic failure) [86].

In conclusion, alpelisib represents a treatment option in the endocrine-resistant management of *PIK3CA*-mutated HR+/HER2– ABC, supported by a PFS benefit, but in the absence of an OS benefit [76, 77]. Fully published trial evidence in the post-CDK4/6i setting still derives, at present, from single-arm phase II data [78]. Capivasertib has been incorporated in the same therapeutic setting for tumors harboring a PI3K-AKT pathway alteration, after demonstration of a PFS benefit in an only partially CDK4/6i pretreated population [82]. While alpelisib and capivasertib exhibit a partially overlapping, class-related toxicity profile, the safety profile reported for capivasertib is considered more favorable, particularly with regard to severe hyperglycemia [77, 82]. It should be clearly emphasized, however, that this observation is based on cross-trial comparison. With PFS benefits shown for both its dual and triplet combinations, gedatolisib emerges as a potential treatment option in the absence of *PIK3CA* mutation [86]. In patients with *PIK3CA*-WT disease, it would have its position as an alternative to previously biomarker-unselected strategies. Though promising, to date, gedatolisib has not yet received Food and Drug Administration (FDA) nor European Medicines Agency (EMA) approval [87, 88]. Across the three trials, fulvestrant served as the control arm, not reflecting the current SOC in most cases. The trials further differed in their pretreatment inclusion criteria—especially relevant with respect to prior fulvestrant and CDK4/6i exposure—and biomarker stratification (Tables 2 and 3); possibly affecting observed treatment effects [76, 82, 86]. The clinical positioning of the ipatasertib-fulvestrant and everolimus-giredestrant combinations within the treatment landscape awaits the full publication of the respective trials [50, 84].

## Non-endocrine-based strategies

### PARP inhibitors

Poly(ADP-ribose)-polymerase inhibitors (PARPi) represent a non-endocrine-based alternative for patients with pathogenic or likely pathogenic germline *BRCA1/2* (*gBRCA1/2*) and somatic *BRCA1/2* (*sBRCA1/2*) mutations, and mutations in germline *PALB2* (*gPALB2*) [89–92].

Two phase III trials tested the PARPi olaparib (OlympiAD) and talazoparib (EMBRACA) against chemotherapy in HER2– ABC in patients with *gBRCA1/2*-mutations with a maximum of three previous chemotherapy regimens and no limit on previous hormonal therapies (EMBRACA) or a maximum of two previous chemotherapies and at least one previous hormonal therapy (OlympiAD). Importantly, these studies were performed before the introduction of CDK4/6i. Both trials reported a significant PFS benefit in the ITT population [olaparib: median PFS 7.0 vs. 4.2 months, HR 0.58 (95% CI 0.43–0.80); *P* < 0.001; talazoparib: median PFS 8.6 vs. 5.6 months, HR 0.54 (95% CI 0.41–0.71); *P* < 0.001], but no improvement in OS was observed [89, 91, 93, 94]. Exploratory subgroup analyses of the respective trials suggested a PFS benefit for olaparib in the triple-negative breast cancer (TNBC) population, but not in HR+/HER2– disease, whereas talazoparib led to improvement in median PFS regardless of subtype [93, 94].

To contextualize the role of PARPi in a treatment landscape after CDK4/6i exposure, a retrospective multi-site analysis suggested a real-world PFS of 8.5 months for talazoparib in HR+/HER2– patients who were mainly pretreated with CDK4/6i [95]. In the phase IIIb LUCY study, no signal of reduced olaparib efficacy was observed in patients with prior CDK4/6i exposure (*n* = 25, median PFS 9.1 months) compared with CDK4/6i-naïve patients (*n* = 12, median PFS 8.4 months), although subgroup sizes were unequal and not formally compared [96].

The phase II TBCRC 048 trial provided a signal of anti-tumor efficacy regarding ORR (primary endpoint) of olaparib in patients with mutations in *gPALB2* (ORR 68% in HR+/HER2– disease, *n* = 19) or with tumors that contain a mutation in s*BRCA1/2* (ORR of 36% in HR+/HER2– disease, *n* = 33). The vast majority of HR+/HER2– patients were CDK4/6i pretreated [92]. No relevant activity was observed for *ATM* or *CHEK2* mutations [90]. Based on these data, testing for *gBRCA1/2* is recommended to guide treatment strategy in HR+/HER2– breast cancer, as is testing for *gPALB2* and *sBRCA1/2,* although with a lower level of evidence and weaker recommendation [13, 14].

### Chemotherapy-based regimens

Chemotherapies, including ADCs, become relevant once endocrine (combination) therapies are exhausted or a rapid tumor response is clinically indicated. Single-agent chemotherapy has been preferred over combination chemotherapy in this setting due to the lack of an OS benefit with combination regimens, except if a rapid response is needed. Generally, taxanes and anthracyclines are the preferred agents, unless there was a recent exposure [13]. Other treatment options include capecitabine, eribulin, vinorelbine, gemcitabine, or platinum agents [13, 14]. Adding bevacizumab to capecitabine or a taxane is an option, having demonstrated a PFS benefit in the absence of an OS benefit [13].

The role of chemotherapy as “first non-endocrine based therapy” has been challenged by the results of the DESTINY-Breast06 trial investigating the anti-HER2 ADC trastuzumab deruxtecan (T-DXd) in HR+/HER2– metastatic breast cancer patients with progression on endocrine-based therapy, including CDK4/6i, but no prior use of chemotherapy for advanced disease. Additionally, tumors were required to demonstrate HER2-low staining assessed by immunohistochemistry (IHC) (IHC score 1+ or 2+ and negative in situ hybridization/ISH) or HER2-ultralow expression (IHC 0 with membrane staining; IHC > 0 and < 1+). Eighty-two percent of patients had HER2-low disease, 89% had prior CDK4/6i exposure, and 41% and 47% had prior anthracycline and taxane therapy, respectively, in the curative setting. T-DXd significantly improved PFS vs. physician’s choice of chemotherapy (capecitabine, nab-paclitaxel, or paclitaxel) in the HER2-low population [primary endpoint, median PFS 13.2 vs. 8.1 months; HR 0.62 (95% CI 0.52–0.75); *P* < 0.001]. ORR was 57% for T-DXd and 32% for chemotherapy in the HER2-low population. The prespecified exploratory analysis of patients with HER2-ultralow disease (18% of patients) showed a numerical trend in PFS in favor of T-DXd [median PFS 13.2 months for T-DXd and 8.3 months for chemotherapy; HR 0.78 (95% CI 0.50–1.21)]. Exploratory analysis of ORR in the HER2-ultralow population was 62% with T-DXd (vs. 26% with chemotherapy) [97]. The three most commonly observed drug-related AEs of any severity with T-DXd were nausea, fatigue, and alopecia; and the most common grade ≥ 3 events were neutropenia (20.7%), anemia (5.8%), and fatigue (3.7%). Drug-related interstitial lung disease (ILD) or pneumonitis occurred in 11.3% of patients (grade ≥ 3 in 1.4%, and grade 5 events in 0.7%); and left ventricular dysfunction/ejection fraction decrease was observed in 8.1% of patients (grade ≥ 3 in 0.7%, no grade 5 events and no reporting of cardiac failure) [97]. Given the pending OS data from this trial, and considering the OS benefit observed in HER2-low disease in DESTINY-Breast04—which evaluated T-DXd after one prior line of chemotherapy—clinicians may currently decide on a case-by-case basis between T-DXd and chemotherapy after CDK4/6i therapy and potentially other endocrine-based therapy lines [98]. In DESTINY-Breast04 and 06, patients with brain metastases were included, but comprised a minority of patients (5–8% of the HR+ population) [97, 98]. An exploratory subgroup analysis of patients with brain metastases (*n* = 24, 75% HR+/HER2– tumors) from the DESTINY-Breast04 trial (presented as an abstract), showed an intracranial ORR of 25% with T-DXd, but no intracranial response in patients treated with single-agent chemotherapy (*n* = 11) [99]. A single-arm phase II trial with 12 patients with more heavily pretreated, HER2-low disease (75% HR+) showed an ORR of 50% in asymptomatic untreated and 33% in patients with progressing brain metastases [100]. Overall, these results indicate that T-DXd demonstrates significant intracranial activity in patients with HR+/HER2-low ABC and brain metastases. Another HER2-directed ADC, BNT323/DB-1303, is under investigation against single-agent chemotherapy in HR+/HER2-low ABC after prior ET and possibly CDK4/6i treatment, but no chemotherapy exposure for advanced disease [101].

In contrast, based on data available in abstract form only, the TROP2-directed ADC sacituzumab govitecan (SG) did not demonstrate a benefit vs. single-agent chemotherapy when being evaluated in the phase III ASCENT-07 trial in patients with HR+/HER2– ABC after progression on ET and mostly prior CDK4/6i therapy, but no previous chemotherapy for advanced disease [102]. The TROP2-directed ADC datopotamab deruxtecan (Dato-DXd) has previously shown a significant PFS benefit, but no OS benefit against chemotherapy of physician’s choice in an HR+/HER2– advanced disease population with progression on 1–2 lines of chemotherapy in the advanced setting and optional prior CDK4/6i exposure (83% of patients) [103, 104]. The TROPION-Breast06 trial is a single-arm phase IIIb trial now evaluating Dato-DXd in advanced HR+/HER2– IHC 0 disease (IHC 0 defined as no staining or incomplete/faint membrane staining in ≤ 10% of tumor cells, reflecting HER2-null or HER2-ultralow status) in chemotherapy-naïve patients after progression on ET [105, 106].

The incorporation of immune checkpoint inhibitors (ICIs) in addition to chemotherapy-based regimens represents an additional strategy under clinical evaluation. The efficacy of ICI therapy in HR+/HER2– breast cancer has been rather disappointing, also reflected in the phase II PACE trial in the post-CDK4/6i setting, in which combining avelumab with palbociclib and fulvestrant failed to improve PFS compared with fulvestrant monotherapy [56, 107]. Adding pembrolizumab to single-agent chemotherapy is being evaluated in the phase III KEYNOTE-B49 trial in PD-L1-positive patients with prior endocrine and CDK4/6i therapy, but no prior chemotherapy for advanced disease [108]. Preliminary data from the phase II SACI-IO trial (in abstract form) compared SG with or without pembrolizumab after mandatory ET pretreatment and in mostly CDK4/6i- and partially chemotherapy-pretreated patients, and showed no significant PFS benefit at a preliminary analysis. Final PFS results are awaited [109]. Similarly, the TROP2–directed ADC sacituzumab tirumotecan is currently being evaluated alone and with pembrolizumab vs. chemotherapy in the phase III TroFuse-010 trial in chemotherapy-naïve patients after progression on endocrine and CDK4/6i therapy [110].

## Emerging therapeutic agents

Beyond the therapeutic strategies discussed above, novel agents with alternative mechanisms of action are under investigation, several of which have already progressed to late-phase clinical trials. The KAT6 inhibitor PF-07248144 is a novel agent in late clinical development targeting the lysine acetyltransferases KAT6A and KAT6B, which are regulators of chromatin organization and transcriptional regulation [111, 112]. PF-07248144 is currently being investigated in combination with fulvestrant in the post-CDK4/6i setting in a randomized, phase III trial [112]. Other investigative efforts have focused on alternative approaches to CDK inhibition beyond CDK4/6i. The more selective CDK4i atirmociclib is under investigation in combination with AI in an advanced first-line setting in phase III trials, directly comparing this approach to the current first-line CDK4/6i standard [31]. Alternative approaches to target CDKs, such as CDK2 or combined CDK2/4/6 inhibition, and novel PROTACs targeting CDK2 are at earlier stages of clinical development [113]. The ADC patritumab deruxtecan, targeting HER3, has shown efficacy in a phase II trial, and is currently also under investigation in a phase III trial in the post CDK4/6i setting [114].

## Discussion

### Clinical decision making

#### General considerations

Therapeutic decision-making in the second-line setting following CDK4/6i progression is complex, owing to several factors: the rapidly expanding therapeutic landscape with multiple emerging options, the absence of direct head-to-head comparisons between available agents, limited cross-trial comparability, which as such has inherent limitations, but is further complicated in this setting by heterogeneous trial populations that may differentially influence treatment effects, the use of suboptimal comparator treatments, the largely absent mature OS data across available trials, fast changes in the first-line and adjuvant treatment standards with direct implications for subsequent therapy and the temporal and methodological aspects of biomarker assessment. In addition, there are unresolved questions of particular complexity, including the management of targetable co-alterations and the question of whether biomarker-selected therapies should be preferred over biomarker-unselected therapies in biomarker-positive patients. Treatment selection is therefore guided by general clinical considerations rather than definite evidence-based recommendations. An overview of the current treatment options with a potential decision tree reflecting the authors’ opinions is provided in Figure 2 and elaborated in the following section, focused on the most recent results from randomized phase III trial data.

Key considerations in patients progressing on ET and CDK4/6i concern, on the one hand, the need for response, which we define as a function of tumor burden with cancer-related symptoms, risk of organ dysfunction including impending visceral crisis, or imminent tumor-related complications. In the presence of a high need for response, prioritizing chemotherapy-based approaches should be considered. On the other hand, decision-making depends on the probability of response to a further non-chemotherapy-based strategy (endocrine-based therapy or PARPi), which may be informed by treatment history (pretreatment duration and response, previous CDK4/6i exposure in more than one setting, prior use of targeted agents or SERDs) and biomarker profile. In addition, disease kinetics play an important role at both levels of this framework: rapid progression may itself indicate a high need for response, and simultaneously inform the assessment of residual endocrine sensitivity. As the general sequencing principle in HR+/HER2– breast cancer favors exhausting ET-based options prior to transitioning to a chemotherapy-based approach [13, 14, 115], a low probability of response does not preclude a further attempt at ET-based therapy in the context of indolent disease.

#### Biomarker-guided strategies

Biomarker status is central to the subsequent treatment choice, but therapeutic decision-making is informed by additional factors and includes the incorporation of the pretreatment history, including tolerability, duration of response and therapeutic agents used in previous treatment lines, disease burden and kinetics, comorbidities and general health condition of the patient, treatment availability and patient preferences. The interplay of these factors ultimately determines the choice between biomarker-guided and biomarker-agnostic strategies. Potential therapeutic strategies stratified by biomarker status are shown in Figure 2.

The interdependence and mutual influence of factors outlined above can be illustrated in the case of an *ESR1* detection. Three agents have shown efficacy in phase III trials as endocrine monotherapy in *ESR1* mutant disease: elacestrant, imlunestrant and vepdegestrant [43, 46, 52]. In this context, the exploratory analysis of elacestrant showing an association of pronounced efficacy with longer prior CDK4/6i duration is cited across guidelines and is reflected, for example, in reimbursement criteria in Switzerland, though the post-hoc design necessitates cautious interpretation [13, 14, 116]. Clinical scenarios in which endocrine monotherapy in *ESR1*-mutated patients may be favored include a low urgency for tumor response—given the consistently steep initial PFS decline observed across all monotherapy regimens—or concerns regarding tolerability of combination regimens [43, 46, 52]. In simple terms, these novel ER targeting agents are an option in patients with *ESR1* mutated disease if 1) achieving tumor stasis rather than regression is sufficient and for 2) low volume disease in which tumor progression does not result in imminent complications such as symptoms and/or organ dysfunction. Furthermore, although imlunestrant and abemaciclib showed superiority over imlunestrant in an unselected patient population in terms of PFS (and in an exploratory subgroup analysis irrespective of *ESR1* mutation status), imlunestrant may be preferred over the combination therapy in *ESR1*-mutated patients in the clinical scenario outlined above—notably the absence of the need for a rapid tumor response (ORR 11% vs. 29% for imlunestrant vs. the combination therapy in ITT, respectively; and ORR of 12% with imlunestrant for *ESR1*-mutated patients in the imlunestrant vs. IC ET arm), poor prior CDK4/6i tolerability; or prior abemaciclib exposure, given the low proportion of abemaciclib-pretreated patients enrolled in the EMBER-3 trial [47]. The position of everolimus and giredestrant within the treatment landscape—for which abstract-presented data have shown a PFS benefit in the ITT population, and pronounced in the *ESR1*-mutated subgroup (both primary endpoints)—remains to be defined pending full publication of the evERA trial [50].

For patients with *PIK3CA*-mutated tumors, both alpelisib-fulvestrant and capivasertib-fulvestrant are effective treatment options. In the absence of head-to-head comparisons, toxicity profiles become a key treatment-selection criterion. The safety profile assessment, based on cross-trial comparison in the absence of head-to-head comparison data, demonstrated higher reporting rates for grade ≥ 3 hyperglycemia with alpelisib than capivasertib (37% and 2.3%), warranting special consideration in patients with pre-existing diabetes mellitus. Grade 3 or higher diarrhea and rash differed less markedly between the two regimens (diarrhea rates of 7% and 9%, and rash rates of 10% and 12% for alpelisib and capivasertib, respectively) [77, 82].

A novel feature in treatment selection is the introduction of the absence of a specific mutation—namely *PIK3CA*-WT status—as a biomarker to guide therapy. This approach is based on proven efficacy of gedatolisib doublet and triplet combinations in a *PIK3CA-*WT population; although direct comparisons of these treatments are lacking, and gedatolisib has not yet received FDA or EMA approval [86–88]. Paradoxically, evidence on the use of gedatolisib-based therapies in *PIK3CA*-mutated disease is yet lacking—a gap expected to be addressed by Study 2 of the VIKTORIA-1 trial, which focuses on a *PIK3CA*-mutated population [86].

With respect to *BRCA1/2*-mutated disease, the evidence supporting PARPi use is heterogeneous and requires consideration for treatment selection. While PARPi have shown efficacy in patients with *gBRCA1/2* mutations, randomized phase III evidence was not derived from a CDK4/6i-pretreated population. Whereas prospective evidence for *sBRCA1/2* and *gPALB2* alterations is derived from mostly CDK4/6i pretreated cohorts (and for olaparib only), it remains confined to studies with limited sample size and ORR as the primary endpoint [90, 92].

#### Biomarker-independent strategies

A biomarker-undefined treatment approach is indicated in the absence of targetable alterations, or if a non-biomarker-guided approach in a biomarker-positive patient is preferred due to clinical or disease-related characteristics.

There are three options to combine a SERD with a CDK4/6i: For fulvestrant-ribociclib and fulvestrant-abemaciclib, treatment effect was demonstrated in populations primarily pretreated with palbociclib. Therefore, the benefit following prior exposure to a different CDK4/6i remains unclear [59, 60]. In the trial evaluating imlunestrant-abemaciclib, only a proportion of patients had received prior CDK4/6 inhibition (predominantly palbociclib), limiting the applicability in the post-CDK4/6i setting [47]. In an exploratory analysis of fulvestrant-ribociclib-treated patients with evaluable ctDNA in the MAINTAIN trial, patients harboring *ESR1* or *PIK3CA* mutations had comparable PFS between the fulvestrant-ribociclib and fulvestrant monotherapy arms, whereas the addition of ribociclib appeared beneficial mainly in mutation-negative patients. Although based on an exploratory analysis with low patient numbers, the analysis represents an attempt to examine the efficacy of biomarker-undirected therapies in biomarker-positive populations [59]. For imlunestrant-abemaciclib, a benefit was consistent regardless of *ESR1* mutation or PI3K-AKT pathway status in prespecified exploratory subgroup analyses [46, 47]. Furthermore, several therapeutic options still originating from the pre-CDK4/6i era remain available, including ET monotherapy with fulvestrant or everolimus-based regimens [13], the latter requiring careful consideration of associated toxicities, including hyperglycemia, pneumonitis or stomatitis [117].

A further unresolved issue is the optimal prioritization of treatment options in the presence of co-alterations. Co-occurrence of *ESR1* and *PIK3CA* for example is consistently reported, with varying prevalence rates [118, 119]. The complexity of co-altered disease may be more directly addressed in clinical trials evaluating combination therapies targeting co-occurring alterations, such as oral SERDs and PI3K/AKT/mTOR-pathway directed agents.

#### Switch to chemotherapy-based regimens

If a chemotherapy-based approach is deemed the most appropriate next therapy line, T-DXd represents an effective option for HER2-low disease, particularly due to its comparatively high ORR of 57%, exceeding the ORR reported for single-agent chemotherapy (32%) in the DESTINY-Breast06 trial [97], and—based on cross-trial comparisons—ORRs achieved by endocrine-based combination therapies from current randomized trials in HER2– populations (ORRs 17–32%) [47, 59, 60, 82, 86]. However, pre-existing cardiac insufficiency or pulmonary comorbidities warrant consideration given its potential cardiotoxic effect and the risk of ILD, and OS data for T-DXd in this setting are still lacking [97]. The evidence base for the use of T-DXd in HER2-ultralow disease derives from an exploratory, prespecified analysis of the DESTINY-Breast06 trial and based on these data, is listed as treatment option in current guidelines [13, 14, 97]. Due to the observed intracranial activity of T-DXd in HER2-low tumors, it might be a preferred agent in patients with brain metastases [99, 100].

### Determinants of biomarker performance

As outlined above, second-line systemic therapy strategies following progression on CDK4/6i have evolved as a biomarker-oriented field, in which therapeutic algorithms incorporate molecular and/or immunohistochemical biomarker testing and one-to-one matching of therapeutic options, which is a consequence of the design from current clinical trials and supports in its simplicity clinical decision making. However, clinical utility of a predictive biomarker might be determined by several, often interrelated considerations.

#### Oncogenic relevance (biological validity)

The oncogenic relevance of the currently clinically relevant biomarkers in second-line HR+/HER2– ABC varies: *ESR1* mutations confer ligand-independent ER activation and represent an acquired resistance mechanism arising under AI therapy [35, 36]*,* whereas *PIK3CA* mutations frequently present as early truncal alterations and a pre-treatment oncogenic driver [64, 72]. HER2-low expression, on the contrary, does not appear to confer relevant oncogenic signal activity in HR+/HER2– breast cancer, but rather serves as a cell-surface target for ADC delivery [120–122].

#### Testing strategy: specimen source, testing timepoint and analytical methods

The likelihood of detecting a biomarker in a tissue biopsy is strongly influenced by intrapatient tumor heterogeneity. An autopsy study from metastatic breast cancer patients, employing multi-platform profiling on multiregional metastases, demonstrated that clinically actionable driver alterations could be truncal and present across metastases, or confined to a subset of lesions. In this study, *PIK3CA* mutations were detected in two patients and were only present in a subset of metastases, whereas *ESR1* mutations were uniformly detected in all metastases in one patient and in only a subset of metastases in another patient [123]. Similarly, substantial intrapatient heterogeneity regarding co-existence of low, ultralow and absence of HER2 expression within the same patient, including in metastases within the same organ, have been demonstrated [124, 125]. These observations illustrate that the detection of a biomarker in a single tissue biopsy does not guarantee its functional dominance across the metastatic landscape. Also, the absence of biomarker detection in a given lesion does not preclude its presence in other lesions.

In this context, ctDNA-based liquid biopsy might represent a complementary approach with the theoretical advantage of analyzing tumor-derived DNA from multiple metastatic sites. However, due to the low concentration of ctDNA in circulation, reliable analysis depends on accurate isolation methods, sensitive assays and careful pre-analytical handling to preserve ctDNA integrity [126].

Studies focusing on concordance rates for *PIK3CA* detection between ctDNA and tissue have reported variable results, possibly due to differences in diagnostic methodology and temporal interval between matched samples. In a study of 72 matched tissue and plasma samples analyzed for predefined *PIK3CA* hotspot mutations with multiplexed PCR-based barcoding of DNA, the concordance rate was 72%, with 14% of mutations detected exclusively in plasma and 8% exclusively in tissue; however, the analyzed samples were not always temporally matched [127]. In a retrospective cohort of patients with tissue and plasma samples taken the same day, concordance rate for three prespecified *PIK3CA* mutations was 100% using BEAMing (PCR-based method). Importantly, in the prospective cohort with a temporal offset between matched samples (median time between tissue and blood sample: 5 years), the concordance rate was lower (73%) [128].

In a recent analysis of a large real-world cohort of breast cancer patients, actionable alterations (including *BRCA1/2, ESR1* and *PIK3CA* mutations) were assessed by next-generation sequencing (NGS) in temporally matched ctDNA and tissue samples. Approximately 59% of alterations were concordantly detected by both modalities, whereas approximately 20% of alterations were identified exclusively by ctDNA, and 21% by tissue profiling. The concordance rates varied between genes; and *PIK3CA* demonstrated the highest concordance (70%) between tissue and ctDNA. For *ESR1*, 65% of variants detected by only one modality were found exclusively by ctDNA [129].

As an additional level of complexity, reliable biomarker detection is contingent not only on the specimen type, but also the timing of testing. Due to the dynamic evolution of *ESR1* mutations under selective pressure of AI [34], testing is indicated on a current sample reflecting post-ET progression status. For *PIK3CA*, mostly considered an early truncal alteration, a retrospective cross-sectional analysis of real-world genomic profiling data from HR+/HER2– breast cancer suggested a relatively stable prevalence of *PIK3CA* mutations at successive lines of therapy in tissue biopsies [118]. In the AURORA program, prospective genomic profiling demonstrated that *PIK3CA* mutations were enriched in metastases compared to their paired primary tumors in mostly treatment-naïve patients, or patients treated with up to one prior line of therapy; however, in the HR+/HER2– subcohort, the rate of *PIK3CA* mutations private to metastasis was low [71]. Furthermore, serial ctDNA sequencing from the PALOMA-3 trial (fulvestrant and palbociclib vs. fulvestrant in an endocrine-resistant population) demonstrated an increase in *PIK3CA* mutation prevalence during treatment. Of the presumably acquired mutations, 33% were detected at low allele frequencies at baseline by droplet digital PCR (ddPCR), suggesting selection of pre-existing subclones alongside treatment-related acquisition of new mutations [73].

Biomarker detection is further influenced by technical and methodological differences among diagnostic assays, including the spectrum of alterations covered and the underlying detection technology. In SOLAR-1 and the VIKTORIA-1 trial, *PIK3CA* mutation status was determined using a PCR test covering predefined hotspot mutations (Table 3) [76, 86]. However, studies with real-world genomic profiling and aggregated genomic datasets suggest that ≥ 20% of pathogenic *PIK3CA* mutations in ABC are not captured by the SOLAR-1 panel, including *N345K*, which might occur more frequently than some included variants [74, 130].

Similarly, different diagnostic approaches for *ESR1* mutation detection are applied across recent studies. In PADA-1, ddPCR provides high sensitivity for hotspot mutations; however, despite covering the majority of actionable mutations, it remains restricted to predefined variants (Table 3) [57, 131]. On the other hand, NGS-based approaches, as applied in SERENA-6 and EMERALD, enable broader detection of *ESR1* variants [41, 45, 131]. These methodological differences can influence the classification of patients as biomarker-positive or -negative, and for *ESR1,* have become particularly relevant when the biomarker serves as marker defining molecular progression prompting a treatment switch [45, 57].

#### Predictive performance of a biomarker

The predictive value of a biomarker and its implementation in clinical decision-making is ultimately determined by evidence derived from clinical trials. Several methodological aspects regarding biomarker-analysis at the trial-design level may potentially influence observed treatment effects with respect to biomarker-defined subgroups. Patients with unevaluable biomarker status can be classified as biomarker-negative, with inconsistent reporting across studies, randomization stratification according to biomarker status can vary, and diagnostic assays differ (Table 3). The definition and assessment of a molecularly defined subgroup can influence the treatment effect in a biomarker-selected subgroup, which was illustrated by the phase II FAKTION trial. Capivasertib plus fulvestrant in AI-resistant disease initially showed benefit over fulvestrant in ITT; with similar effect sizes observed irrespective of PI3K-AKT pathway alteration status (including *PIK3CA* and *PTEN* alterations). However, refined post-hoc biomarker analysis using an extended set of molecular alterations (including *AKT1*), and more sensitive diagnostic assays, resulted in substantial patient reclassification. The new analysis suggested that clinical benefit was confined to pathway-altered tumors, with no signal of efficacy observed in biomarker-negative disease, underscoring the importance of precise molecular stratification [132, 133].

In addition, some phase III clinical trials investigating treatment effects according to biomarker status incorporate a dual-testing framework comprising a biomarker-unselected and a biomarker-selected population. Biomarker-negative populations are then investigated in an exploratory setting, which may reduce the likelihood of detecting treatment effects in this subgroup.

Study 1 of the two-part VIKTORIA-1 trial takes a novel, methodologically distinct approach by assessing treatment efficacy in a specifically mutation-negative population: The PI3K/mTORC1/2 inhibitor gedatolisib has demonstrated activity in an exclusively *PIK3CA*-WT population, providing more robust evidence especially for patients without actionable alterations [86]. As gedatolisib additionally functions as an mTORC inhibitor, the observed treatment effect may be mediated through mTOR inhibition, which acts downstream of PI3K and for which patient selection has not been biomarker-driven [86]. In study 2 of VIKTORIA-1, focusing on the *PIK3CA*-mutated cohort, the gedatolisib combination therapies will be compared to alpelisib and fulvestrant and will provide further insights into the clinical significance of *PIK3CA* with respect to gedatolisib activity [134].

The evolving understanding of the predictive value of biomarkers is illustrated by T-DXd and HER2 expression; initially restricted to HER2-positive patients [135], it has also shown clinically relevant efficacy in HER2-low disease and has shown signals of activity even in HER2-ultralow tumors [97].

#### Implications for clinical practice

Due to the spatial and temporal heterogeneity, and variability in analytical methods, recommendations for the assessment of biomarkers in clinical practice differ between individual biomarkers. Regarding *PIK3CA*, *AKT1* and *PTEN* testing, current guidelines either mostly do not address whether ctDNA or tissue should be preferred, or list both options without hierarchy. Similarly, no clear preference is established with respect to timing (archival vs. current sample) or lesion type (primary vs. metastatic site) [13, 14, 136]. Of note, NCCN Guidelines^®^ recommend to prefer tissue-based testing over ctDNA to detect *PTEN* homozygous copy loss [14]. Today, *PIK3CA* mutation status is the only biomarker required already before the start of a first-line palliative therapy in case of recurrence during or shortly after adjuvant ET based on the results of the INAVO120 trial [26]. With respect to *ESR1* mutations, given the dynamic acquisition of *ESR1* mutations under ET, current guidelines recommend testing for *ESR1* at time of progression on ET, preferably with ctDNA or, alternatively, within a tumor tissue sample [13, 14, 137]. In contrast, the determination of HER2-low status is IHC-based and thus only assessable in tissue. Guidelines acknowledge the temporal and spatial heterogeneity of HER2 expression and recommend a repeat biopsy or the integration of results from all available prior and concurrent samples, where this may inform clinical decision-making [13, 14, 138]. Regarding diagnostic assays, the ESMO Precision Medicine Working group advocated 2024 for NGS as SOC; however, this recommendation predates the introduction of inavolisib in the first-line palliative therapy; and does not account for the divergent timepoints of the disease course at which *PIK3CA* and *ESR1* mutations are required [139].

In summary, in the post-CDK4/6i setting, the highlighted multifaceted variables of therapeutically actionable biomarkers ask for a personalized strategy on a case-to-case basis, but in general requires the determination of predictive biomarkers on a freshly obtained blood (ctDNA) or tissue sample, due to the acquisition of *ESR1* mutations during ET. As a rule of thumb, we favor ctDNA testing in cases of difficult-to-access lesions or widespread tumor progression. A fresh tissue biopsy could be considered suitable in cases of isolated progression (with the goal to capture the molecular landscape of the progressive lesion); and/or when a switch to a chemotherapy-based regimen is clinically indicated and prior HER2 assessments have indicated HER2-null status (IHC 0 with complete absence of membrane staining, thus not meeting the criteria for HER2-ultralow) [106].

## Conclusion

The treatment landscape of HR+/HER2– ABC in the post-CDK4/6i setting has become increasingly complex, with a growing number of therapeutic options but no standardized treatment algorithms. Treatment selection therefore requires an individualized approach integrating patient- and disease-related factors. While biomarker-guided strategies are central to guide treatment decisions, their optimal clinical implementation requires careful evaluation of their biological, contextual and methodological complexity.

The prevailing singular biomarker-driven approach appears insufficient to fully capture context-dependent disease biology and its associated heterogeneity in treatment response. With the fast pace of emerging new therapies, it becomes increasingly difficult to design clinical trials focusing on unmet needs, such as therapy sequencing or prioritizing of biomarker-uninformed vs. -informed approaches, which are still of clinical value at completion. There is a need for a more holistic profiling strategy that accounts for individual disease biology and enables the prediction of therapeutic susceptibility in different clinical scenarios.

A suggested strategy is represented by precision oncology focused trials using multi-omics profiling and functional drug screening [140] resulting in patient allocation to clearly predefined treatment strategies. Along these lines, the LINUX trial pioneered this approach with reclassification of HR+/HER2– breast cancer using a multi-omics approach, translation of these classifications into artificial intelligence-assisted digital pathology methods and as a result, patient allocation to different treatment arms [141]. Complementary to these efforts, a deeper mechanistic understanding of sensitivity and resistance to ET-based treatment strategies may be advanced through cutting-edge translational research programs accompanying clinical trials with patient avatars (patient-derived xenograft or organoid models) to model treatment resistance and repeat biopsies upon disease progression for transcriptomic, genomic and (phospho)proteomic analysis, followed by functional assays to validate the causality of the obtained correlative results. Collaboration between research laboratories and oncologists should be fostered to ensure that mechanistic research remains responsive to emerging developments and unmet needs in medical oncology. In the context of unresolved clinical questions, existing real-world data containing available molecular tumor profiles hold substantial potential for hypothesis-generating findings regarding, e.g., therapy sequencing and association of therapy response with somatic molecular alterations beyond those covered in clinical trials.

Clinical practice is shaped by phase III trial results and depends on trial design allowing for successful translation into routine care. Benefits in PFS observed in clinical trials have constituted the predominant measure of efficacy of investigated treatment strategies. Demonstration of OS benefit is hampered by the requirement for prolonged follow-up and possible inherent dilution effect imposed by the continuously changing treatment landscape and subsequent treatment lines. Given these limitations, the incorporation of patient-centered and/or patient-defined endpoints into trial design assumes increasing importance, such as health-related quality of life or preservation of functional independence.

## Abbreviations

ABC: advanced breast cancer
ADC: antibody-drug conjugate
AE: adverse event
AI: aromatase inhibitor
ALT: alanine aminotransferase
AST: aspartate aminotransferase
CDK4/6i: CDK4/6 inhibitor(s)
CDK4i: CDK4 inhibitor(s)
CERANs: complete estrogen receptor antagonists
CI: confidence interval
ctDNA: circulating tumor DNA
Dato-DXd: datopotamab deruxtecan
EMA: European Medicines Agency
ER: estrogen receptor
ET: endocrine therapy
FDA: Food and Drug Administration
*gBRCA1/2*: germline *BRCA1/2*
*gPALB2*: germline *PALB2*
HER2–: HER2-negative
HR: hazard ratio
HR+: hormone receptor-positive
IC ET: investigator’s choice of endocrine therapy
ICIs: immune checkpoint inhibitors
iDFS: invasive disease-free survival
IHC: immunohistochemistry
ILD: interstitial lung disease
ITT: intention-to-treat
NGS: next-generation sequencing
ORR: overall response rate
OS: overall survival
PARPi: Poly(ADP-ribose)-polymerase inhibitor(s)
PFS: progression-free survival
PROTAC: proteolysis-targeting chimera
*sBRCA1/2*: somatic *BRCA1/2*
SERD: selective estrogen receptor degrader
SERMs: selective estrogen receptor modulators
SG: sacituzumab govitecan
SOC: standard of care
T-DXd: trastuzumab deruxtecan
TNBC: triple-negative breast cancer
WT: wild-type

## Declarations

### Copyright

© The Author(s) 2026.
